# Development of a highly degenerate primer-based molecular tool for detecting and classifying the four major classes of polyhydroxyalkanoate synthase (*phaC*) genes in bacteria

**DOI:** 10.1186/s12934-025-02831-9

**Published:** 2025-09-22

**Authors:** Abdiqani Ibrahim Osman, Brendon Noble, Linda Percy, Pooja Basnett

**Affiliations:** Sustainable Biotechnology Group, Centre for Nutraceuticals, School of Life Sciences, College of Liberal Arts & Sciences, University of Westminster, London, W1W6UW UK

**Keywords:** Marine bacteria, Polyhydroxyalkanoate, Bioplastics, Molecular screening

## Abstract

Polyhydroxyalkanoate synthase (*phaC*) gene encodes for PHA synthase enzyme which plays a key role in PHA polymerisation. To screen, unknown bacterial strains for their potential to produce PHAs, the presence of *phaC* gene is essential. Currently published primer sets targeting *phaC* gene are inadequate and often work only for well-studied genera (e.g., *Pseudomonas*, *Cupriavidus*). Few studies validate them in vitro, even fewer use degenerate primers to address *phaC* sequence diversity, and many fail to target all four *phaC* classes. In this study, nine novel highly degenerate primers were designed using the HYDEN (HighlY DEgeNerate) tool. The design included 65 *phaC* gene sequences from class I, 10 from class II, 19 from class III, 30 from class III/IV, and 6 from class IV, carefully selected as a representative sample size to capture the variations among bacterial strains and *phaC* sequences. The primer specificity was then assessed in silico with De-MetaST-BLAST against all known *phaC* sequences in the NCBI database. This was followed by in vitro screening of seven bacterial strains known to express the four major classes of *phaC* genes and 15 novel marine bacterial strains in which *phaC* presence were unknown. Seven strains—namely *Halomonas alkaliphila* DINO, *Marinobacter* sp. MB2, *Halomonas profundus* NQ7, *Halomonas titanicae* MC2, *Bacillus pacificus* C4, *Bacillus pacificus* B4 and *Bacillus mycoides* B12 tested positive. All 15 strains were subjected to nutrient limiting growth conditions to assess PHA production with results confirming molecular screening. This study demonstrates the successful development and validation of a highly degenerate primer-based molecular screening tool capable of detecting and differentiating the four major classes of *phaC* genes in well-known non-marine and novel marine PHA-storing bacteria.

**Graphical Abstract:**

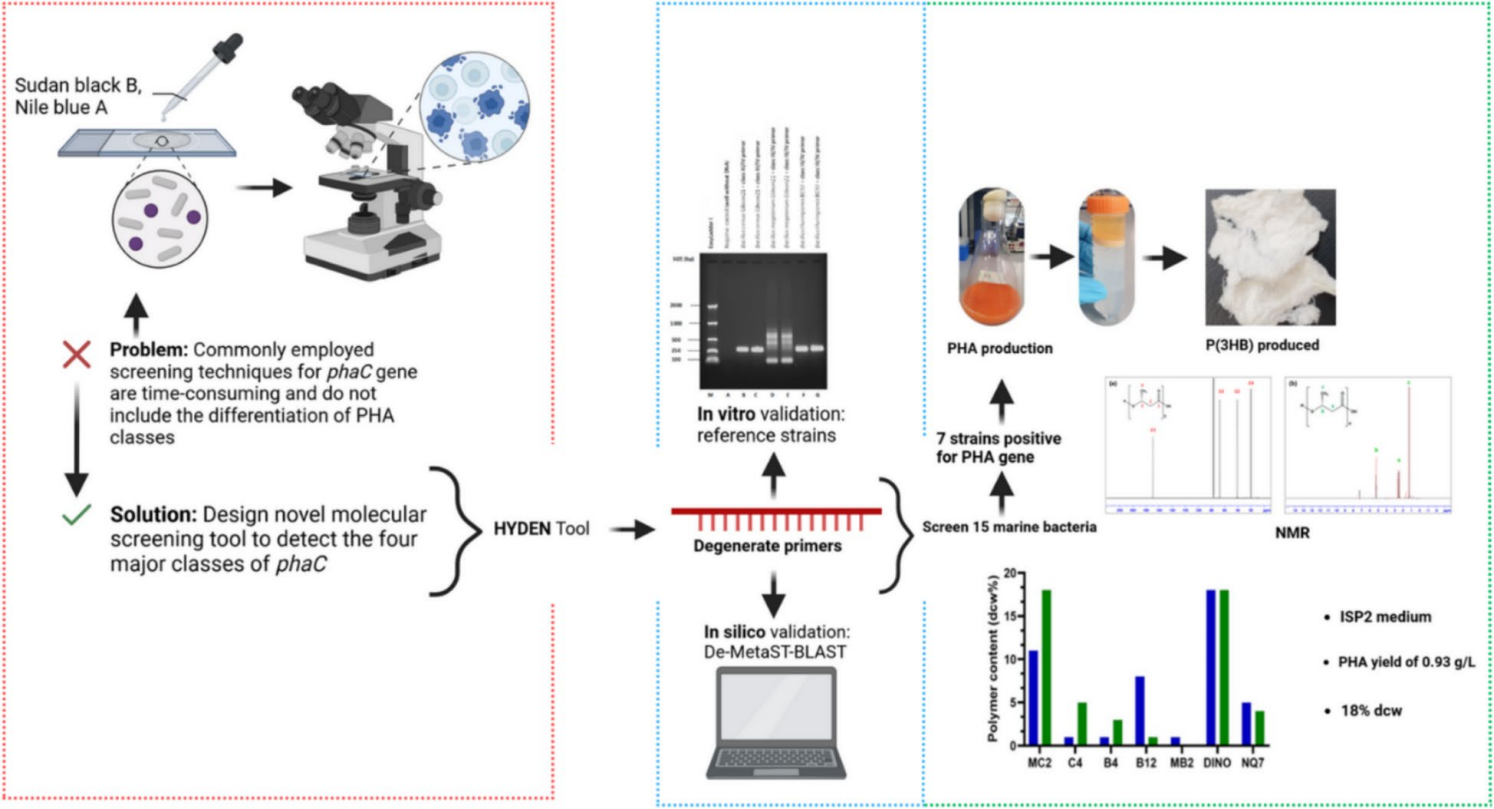

**Supplementary Information:**

The online version contains supplementary material available at 10.1186/s12934-025-02831-9.

## Highlights

- Nine highly degenerate primers were designed using the HYDEN primer tool and validated in silico with De-MetaST-BLAST against all known *phaC* sequences in the NCBI database and validated in vitro using known PHA producers.
- 15 novel marine bacterial strains were screened for the presence of *phaC* gene using the nine degenerate primers. Seven strains - namely *Halomonas alkaliphila* DINO, *Marinobacter* sp. MB2, *Halomonas profundus* NQ7, *Halomonas titanicae* MC2, *Bacillus pacificus* C4, *Bacillus pacificus* B4 and *Bacillus mycoides* B12 tested positive.
- To assess whether the presence of the *phaC* gene serves as a reliable indicator of PHA accumulation, both *phaC* screening-positive and *phaC* screening-negative strains were subjected to nutrient-limited growth. NMR analysis confirmed the production of short-chain-length P(3HB) in positive strains.
- *Halomonas alkaliphila* DINO produced the highest PHA yield of 0.93 g/L and an intracellular content of 18% dry cell weight when cultivated in an ISP2 medium.

**Supplementary Information:**

The online version contains supplementary material available at 10.1186/s12934-025-02831-9.

## Introduction

Plastics, a global staple, have been produced from non-renewable fossil fuels since the 1940s, with over 8.3 billion metric tonnes produced so far [13, 17]. These plastics, such as Polyethylene (PE), Polyethylene Terephthalate (PET) and Polyvinyl Chloride (PVC), are used extensively in industries, however, they are unsustainable due to short life spans, leading to significant plastic waste accumulation [15, 17, 51]. Landfilling, incineration, and recycling are methods for managing plastic waste, but there are limitations such as greenhouse gas emissions, generation of microplastics and downcycling where the recycled material is of lower quality and functionality than the original material [17]. To mitigate these issues, several measures such as single-use plastic prohibition, carrier bag levy, and recycling infrastructure enhancement have been implemented in some countries [21]. However, to address plastic pollution, it is necessary to transition to environmentally sustainable alternatives.

Polyhydroxyalkanoates (PHAs) are sustainable, natural polymers produced by a variety of Archaea and Gram-positive and Gram-negative bacteria [23, 37, 65]. In recent times, marine bacteria have been explored for the production of PHAs due to their resistance to contamination, growth at low temperature and fast-growing ability [20, 47, 57, 59, 63]. In the organism, PHAs serve as carbon and energy storage molecules, exist in water-insoluble granules, and represent up to 90% of cell dry weight [35, 60]. PHAs contain around 140 distinct hydroxyalkanoic acids, accumulated by microbial species from over 90 genera [9, 56]. PHAs, such as poly(3-hydroxybutyrate (P3HB), poly(3-hydroxybutyrate-co-3-hydroxyvalerate (PHBV), poly(3-hydroxybutyrate-co-4-hydroxybutyrate (P3HB4HB), and poly(3-hydroxybutyrate-co-3-hydroxyhexanoate (PHBHHx), are used in various industries such as packaging films and 3D printing [65]. PHAs also possess biodegradability and biocompatibility, rendering them suitable for deployment in medical implants, including soft tissue engineering such as cardiovascular, neural or in wound healing applications [6, 14, 22]. However, they face challenges such as high production costs, low productivity, and unstable product quality [58].

The classification of genes involved in PHA synthesis is based on their gene locus organisation, as well as substrate preference and subunit composition of the enzymes [9, 66]. Polyhydroxyalkanoate synthase (*phaC*) gene encodes for PHA synthase enzyme which plays a key role in PHA polymerisation. They are distinguished by a preserved lipase-like box (G-X-S-X-G) and by three preserved amino acids cysteine, aspartic acid, and histidine (CDH) that make up the *phaC* catalytic triad [10, 14, 31]. Four classes (i.e., class I, II, III and IV) of *phaC* are known, each with their distinct characteristics and substrate specificity which determines the types of monomers to be included into the PHA polymer [9, 66]. The class I PHA accumulating organisms such as *Azotobacter chroococcum* all have the pathway consisting of a ketothiolase locus (*phaA*), dehydrogenase (*phaB*) and polyhydroxyalkanoate synthase (*phaC*) [9, 40]. The class II locus, largely found in *Pseudomonas* species, consists of an operon with two PHA synthase genes (*phaC1* and *phaC2*) on either side of a PHA depolymerase gene. The class II *phaCs* are found in medium-chain-length PHA producers, that rely on beta oxidation or de novo fatty acid synthesis for PHA accumulation [9]. In certain *Bacillus* species, such as *Bacillus thuringiensis*, the class III operon includes the *phaC*, *phaA*, and *phaB* genes, similar to class I operons, and is responsible for the production of short-chain-length PHAs [9, 66]. The presence of class IV PHA loci, consisting of *phaR* and *phaC* genes encoding the two hetero-subunits of the active PHA synthase, was first discovered in the bacterium *Priestia megaterium* (formerly known as *Bacillus megaterium*) [9, 66]. The length of the PHA operon varies according to the class, with class I ~ 3684 bp, class II ~ 5753 bp, class III ~ 15100 bp, and class IV ~ 3396 bp (Rehm, 2003). Given their genetic diversity and biotechnological potential, PHAs are gaining commercial interest, with global production projected to reach 7.6 million tonnes by 2026 [33]. Therefore, there is a huge interest around finding novel strains with PHA production capabilities.

Screening bacterial cultures for the presence of PHA gene requires the use of efficient, straightforward, quick, and dependable methods. Commonly employed screening techniques encompass the utilisation of Sudan black B, Nile blue A, and Nile red, which can be directly introduced into cell cultures [9]. However, some of these methods are time-consuming and do not include the differentiation of PHA classes. Thus, alternative screening techniques such as Fourier transform infrared spectroscopy (FT-IR) and the polymerase chain reaction (PCR)-based methods can be employed since they offer benefits such as simplified sample preparation, cost-effectiveness, reduced labour requirements, and rapid analysis [9]. Several studies have reported primer sets for *phaC* synthase gene, as summarised in Table 1.

**Table 1:** Review of primer sets targeting PHA synthase (*phaC*) gene and their limitations

Primer details	Target genes	Key findings/limitations	References
Designed three degenerate primers for amplifying fragments from *phaC* (Class I), *phaC1*, and *phaC2* (Class II) genes	*phaC* (Classes I and II)	The study focused on Gram-negative bacteria, which may limit its applicability to Gram-positive PHA producers	[50]
Designed primers based on unique regions of the *phaC* gene of *Bacillus megaterium*	*phaC* (Class IV)	The primers are specific to *B. megaterium* and may not be applicable for detecting *phaC* genes in other bacterial species	[36]
Designed three degenerate primers based on multiple sequence alignment	*phaC* (classes not considered)	The study did not explore the detection of different classes of *phaC* genes and the primer sets mainly target the genus *Pseudomonas*	[52]
Developed a self-designed primer set (phaCF1BO/phaCR2BO) and compared it with a previously reported set that mainly target the genus *Pseudomonas* designed by Sheu et al.[52]	*phaC* (Classes I and II)	The study focused on activated sludge samples, which may limit the applicability of the findings to other environmental samples. Additionally, the detection was limited to classes I and II PHA synthases	Tzu and Semblante [64]
Designed primers based on a wide variety of *phaC* gene sequences	*phaC* (Classes I and IV)	The study did not address the detection of *phaC* gene from classes II and III, potentially limiting its applicability to a broader range of PHA-producing bacteria	[32]
Designed primers based on *phaC* gene sequences within Cyanobacteria	*phaC* (classes not considered)	The study did not explore the detection of different classes of *phaC* gene, and the primer sets mainly target *phaC* gene within Cyanobacteria	[25]

However, key observations from literature include, restricted number of sequences used to design primer sets, primers often work only for well-studied genera (e.g., *Pseudomonas*, *Cupriavidus*), few studies validate primers experimentally, limited studies use degenerate primers to account for *phaC* sequence diversity and many primer sets fail to target all four *phaC* classes. In this study, we have designed highly degenerate primer sets using the HYDEN tool and validated their specificity and coverage in silico. De-MetaST-BLAST was used to assess target matching against all known *phaC* sequences in the NCBI database, while the Eurofins Genomics Oligo Analysis Tool evaluated primer properties such as GC content, melting temperature, and secondary structures. PHA production was then carried out through flask cultivation, with results confirming the findings from the molecular screening, thereby ruling out false positives. This study demonstrates the successful development and validation of a highly degenerate primer-based molecular screening tool capable of detecting and differentiating the four major classes of *phaC* genes in well-known non-marine and novel marine PHA-storing bacteria, thus supporting their application in PHA production.

## Materials and methods

### Chemicals

All chemicals were procured from Merck (Darmstadt, Germany), Thermo-Fisher Scientific (Dartford, UK), Scientific Laboratory Supplies Ltd (Wilford, UK), Eurofins Genomics (Wolverhampton, UK), and Qiagen (Manchester, UK). Bacterial culture media were obtained from Sigma-Aldrich (Dorset, UK). All studies were conducted using distilled or analytical grade reagents. Primers for PCR were synthesised by Eurofins custom DNA oligos (https://eurofinsgenomics.eu/en/dna-rna-oligonucleotides/custom-dna-rna-oligos/custom-dna-oligos/). DNA extraction, PCR and gel purification kits were purchased and obtained from Qiagen Ltd. Difco Marine Agar 2216 were sourced from Thermo-Fisher Scientific. Millipore Nutrient Broth 70122, Millipore Nutrient Agar No 2 7116 and Millipore Marine broth 2216 were sourced from Sigma-Aldrich.

### Bacterial strains, maintenance and growth medium

To validate the molecular screening tool, confirmed polyhydroxyalkanoate (PHA)-producing bacterial strains representing the four major *phaC* classes were obtained from the National Collection of Industrial and Marine Bacteria (NCIMB), Aberdeen, UK. Strain selection was informed by prior literature and corresponding *phaC* gene annotations available in the NCBI database. Details of the selected reference strains along with their confirmed *phaC* gene classes are provided in Table 2. With the exception of *Azotobacter chroococcum*, all bacterial cultures were revived, maintained, and grown on Millipore Nutrient Broth 70122 (composition: D ( +)- glucose, 1 g/L, peptone 15 g/L, sodium chloride 6 g/L and yeast extract 3 g/L) and Millipore Nutrient Agar No 2 7116 (composition: agar 15 g/L, meat extract 3 g/L and peptone 5 g/L) at 30ºC and 120 rpm for 24 h. *Azotobacter chroococcum* was revived and cultivated using the specific media (composition: agar 15 g/L, distilled water 950 ml, glucose 5 g/L, mannitol 5 g/L, CaCl_2_xH_2_O 0.10 g/L, MgSO_4_ × 7H_2_O 0.10 g/L, Na_2_MoO_4_ × 2H_2_O 5 g/L, K_2_HPO_4_ 0.90 g/L, FeSO_4_ × 7H_2_O 0.01 g/L, CaCO_3_ 5 g/L) obtained from MediaDive database accessible from (https://mediadive.dsmz.de/) [24]. Glucose and mannitol were sterilised separately in 50 ml distilled water and added to the medium after autoclaving and pH adjusted to 7.3. All cultures were stored on agar plates at + 4 ºC and kept in cryovials at −20 ºC and −80 ºC in a 40% glycerol solution.

**Table 2:** Bacterial strains used for in vitro validation of degenerate primers

Strain	PHA class	NCIMB ID
*Azotobacter chroococcum*​	I	NCIMB10427
*Pseudomonas mendocina* CH50​	II	NCIMB10541
*Bacillus thuringiensis​* BC93	III	NCIMB8705
*Bacillus megaterium Gibson21*	IV	NCIMB4821
*Bacillus cereus*	IV	NCIMB13123
*Bacillus cereus Gibson21*	IV	NCIMB7587
*Priestia megaterium*	IV	NCIMB8674

### Isolation and identification of a novel marine bacteria

The marine bacteria cultures were all isolated as a part of the Antibiotics Unearthed project led by Dr Linda Percy. Samples were obtained from marine sediments collected at various UK coastal sites, including Weymouth (strains *Bacillus hwajinpoensis* A9, *Bacillus pacificus* C4, *Bacillus pacificus* B4, *Bacillus mycoides* B12 and *Pseudalkalibacillus hwajinpoensis* B9), Port William (*Bacillus stercoris* F2, *Bacillus pumilus* PW3 and *Cytobacillus firmus* PW2), Montrose (*Halomonas titanicae* MC2, *Halomonas profundus* NQ7, *Marinobacter sediminum* MB2 and *Pseudalkalibacillus hwajinpoensis* BS1), Holy Island (*Pseudalkalibacillus hwajinpoensis* LY) and Bristol (*Pseudalkalibacillus hwajinpoensis* B80). Additionally, *Halomonas alkaliphila* DINO was isolated from an association with the microalgal species *Amphidinium carterae*. Marine bacteria were cultured as described by Percy et al. [43]. Upon collection, sediment samples were stored at 4 °C. Later, 5 g of marine sediment was added to Erlenmeyer flasks with 900 mL of 0.2 μm Filtered (Nucleopore) and Autoclaved SeaWater (FASW). To inhibit microalgal growth, flasks were wrapped in foil and incubated at 18 °C with agitation at 120 rpm. Over a 2-week period, samples were taken weekly, serially diluted to 10^–7^ in FASW, and 100 μL aliquots were spread on 50% diluted Difco Marine Agar 2216 supplemented with 2% agarose (Thermo-Fisher Scientific). Plates were incubated at 18 °C until discrete colonies formed. Individual colonies were then isolated using sterile wire loops, streaked onto fresh marine agar, and incubated at 18 °C. Each isolate was re-streaked at least three times to obtain pure cultures. Finally, the bacterial strains were identified through amplification of the 16S rDNA gene using universal forward primer 27F 5′ AGA GTT TGA TCM TGG CTC AG 3’ and reverse primer 1492R 5′ CGG TTA CCT TGT TAC GAC TT 3’ [49]. The PCR conditions and DNA extraction method is summarised in "Genomic DNA extraction, PCR amplification and 16S rDNA gene sequencing" section. Annealing temperature used was 51 °C.

### Retrieval of PHA sequence and library construction

A nucleotide search was conducted for the complete sequence of *phaC* genes against NCBI database (https://ncbi.nlm.nih.gov) from which a total of 65 class I, 10 class II, 20 class III and 6 class IV were downloaded in FASTA format (see the supplementary material). The majority of sequences selected were from different bacterial strains known to express *phaC* synthase gene in the phyla Proteobacteria and Firmicutes respectively because of the availability of data.

### Design and evaluation of primers for the amplification of the *phaC* gene

The protocol for primer design was similar to that described by Chukwuemeka et al. [11]. The primers were developed using multiple software programmes and web servers such as Highly Degenerate primer (HYDEN) programme [27] accessible from (http://acgt.cs.tau.ac.il/hyden/hyden_license.html), Molecular Evolutionary Genetics Analysis version 11 (Tamura, Stecher, and Kumar 2021) accessible from (https://megasoftware.net/), De-MetaST and De-MetaST-BLAST [18] available at (http://sourceforge.net/p/de-metastblast/) and (http://code.google.com/p/de-metast-blast/). The HYDEN command-line parameters used can be found in the supplementary material. Both De-MetaST and De-MetaST-BLAST has been developed for Ubuntu operating systems. Thus, a Virtual Machine (VM) with Linux distribution software Ubuntu 22.4 with RAM 16 GB, 4 VCPU and a storage of 500 GB was used on a Dell OptiPlex 7020 personal desktop computer. The computer benchmarks were composed of an Intel i5-4590 CPU @ 3.30 GHz, 3300 MHz, 4 Core, Microsoft Windows 10 Pro, × 64-based, RAM 8 GB, and a storage of 500 GB.

### In silico analysis of degenerate primers

The primers were procured from Eurofins Scientific and received in a lyophilised state and diluted using DNase/RNase free molecular water to a working solution of 20 pmol/μL and stored at a temperature of −20 °C. To assess the suitability of the degenerate primer pairs for PCR, thermodynamic properties and self-complementarity tests were performed using the online Eurofins genomics Oligo Analysis Tool accessible from (https://eurofinsgenomics.eu/en/ecom/tools/oligo-analysis/). This programme automatically assessed primers for several characteristics such as max 3’ annealing score, max 5’ annealing score, Tm difference and provides recommendation for use in PCR. In addition, the nucleotide sequences of *phaC* were aligned utilising MUSCLE in MEGA 11 software to ascertain the specific locations where the primers exhibit binding affinity. This analysis also enabled the estimation of the size of the amplicons and the consensus position. In addition, bioinformatics tool known as De-MetaST in conjunction with BLAST was used to assist in validating the Degenerate Primer Design (DPD) specificity.

### Genomic DNA extraction, PCR amplification and 16S rDNA gene sequencing

Bacteria were grown in respective sterile media broth at 30 ºC. To ensure complete dissolution of the Marine agar 2216 and broth (Millipore Sigma-Aldrich, Dorset, UK), the solution was heated and agitated on a hotplate before subjecting it to autoclaving at a temperature of 121 °C for a duration of 15 min. DNA was extracted from 24 h old culture of ~ 2 × 10^9^ cells as per the Qiagen DNeasy blood and tissue kit protocol. The quality and concentration of nucleic acid was assessed by the 260/280 ratio of absorbance wavelengths using Nanodrop One Microvolume UV–Vis spectrophotometer from Thermo Fisher Scientific, Loughborough, UK, requiring 1 μL of isolated nucleic acid for analysis. A 260/280 ratio of ~ 1.8 is generally accepted as high-quality DNA [62]. PCR was first used to assess primer specificity and then to confirm the presence of the target gene in DNA samples extracted from reference and marine bacteria collections. The 25 µL reaction mixture contained 4 µL of genomic DNA as template (concentration 11–25 ng/µL), 0.5 µL of each primer (final primer concentration 20 pmol/µL), 12.5 µL MyTaq Red Mix Bioline Meridian and 7.5 µL molecular grade water. All reactions were carried out in a thermocycler (Bio-Rad T100) consisting of initial denaturation at 95 ºC for 1 min, followed by 35 cycles of denaturation at 95 ºC for 15 s, annealing at (optimised using gradient temperatures) for 15 s and final extension at 72 ºC for 10 s. Novel bacterial strains were identified by the amplification of 16S rDNA gene followed by Sanger sequencing and NCBI BLAST analysis.

### Gel electrophoresis for the detection and validation of PCR amplicons

Electrophoresis on 1% (w/v) precast agarose gels from Sigma-Aldrich was employed. Meridian Bioscience HyperLadder 25 bp—100 Lanes, Meridian Bioscience EasyLadder 1 50–2000 bp and Sigma-Aldrich P9577 PCR 50–2000 bp ladder were used as DNA size makers. Run conditions were 65 V for 90 min in 1X Tris–borate-EDTA buffer (composition 89 mM Tris, 89 mM boric acid, 2 mM EDTA). Amplified DNA fragments were visualised and recorded using UVP BioDoc-It Imaging System UV Transilluminator. Amplified products from bacterial strains screened having expected amplicon size on the gel image were purified using Qiagen Aquick PCR & Gel Cleanup Kit following manufacturers protocol. Sequencing was carried out by Eurofins Genomics, UK, using both forward and reverse primers listed in Table 3. The raw sequence data and chromatograms were quality-checked using SnapGene accessible from (https://www.snapgene.com/) and sequences were aligned using MUSCLE in MEGA 11. Subsequent comparisons were performed against the NCBI nucleotide database using both BLASTn and BLASTx.

**Table 3:** Sequence of degenerate primers designed and used in this project

Primer ID	Class	Sequence^x^ (5’-3’)	Degene-racy^y*^	Amplicon size (bp)	Coverage^z^	Tm [˚C]	Ta [˚C]	GC- content (%)
*phaCF1* *phaCR1*	I I	ATCAAYAAGTAYTACATYCT TTCCAGWASARCAKRTC	14 32	558	52/65 48/65	48.1 49.2	44	28 44
*phaCF2* *phaCR2*	I I	BSARYTSMTYCARTAYVV TCNARRATRTARWAYYKRTT	2048 4608	92	62/65 62/65	51.4 48.1		44 28
*phaCF3* *phaCR3*	II II	CTGGATGCGCCCCAACGA TCSAYCGGCGTRCCRCACA	1 48	203	8/10 10/10	60.5 62.1	58	67 66
*phaCF4* *phaCR4*	III III	CTKRTYAAYMRNCCVTAYAT CARTCNADCABVYASACRTC	1536 1728	94	17/19 18/19	52.5 56.6		38 48
*phaCF5* *phaCR5*	III III	GAHCATATTTCTAGCACAGA CCCCARTCNADCABRTAC	16 144	708	12/19 17/19	51.8 54.8	46	37 53
*phaCF6*	IV	TATCGTCARTGGATTCGHGA	2	305	5/6	54.9		44
*PhaCR6*	IV	AAATCAAAHGGASWWGTCATAAA	6		5/6	52.3		28
*phaCF7*	IV	GATGATTGAYTTTGGAAABAA	8	421	12/13	50.4		29.4
*PhaCR7*	IV	GTTAAATCNARAATATACGG	2		11/13	49.1		30
*phaCF8* *PhaCR6*	III & IV III & IV	GTGTAYNTGYTDGAYTGGGG AAATCAAAHGGASWWGTCATAAA	96 6	244	12/13 11/13	58.0 52.3	50	28 52
*phaCF8* *phaCR8*	III & IV III & IV	GTGTAYNTGYTDGAYTGGGG GGRATRTTKCCVARYGTATC	96 64	352	26/30 24/30	58.0 55.6	50	52 46

^x^Nucleotide bases other than the standard Watson–crick bases depict the universal codes for degenerate bases or "wobbles": R = A/G, Y = C/T, M = A/C, K = G/T, S = C/G, W = A/T, B = C/G/T, D = A/G/T, H = A/C/T, V = A/C/G, and N = A/C/G/T

^y^In order to amplify as many input sequences as possible and additional novel genes within the gene superfamily, the Hyden software incorporates degeneracies within the primers

^z^The coverage shows what proportion of the input sequences are covered by the two primer

^*^Allowing a maximum of two mismatches (one mismatch in 5' end and one mismatch in 3’ end)

### PHA production via flask cultivation using novel marine bacterial strains

PHA production via flask cultivation was performed using 15 novel marine bacterial strains, including both *phaC* screening-positive (n = 7) and *phaC* screening-negative (n = 8) cultures (Table 4). 25 mL of sterile marine broth 2216 was inoculated separately with each strain. The cultures were then incubated for 24 h at 30 ºC and 130 rpm. The seed cultures were transferred into 500 mL shaken flasks with 225 mL of the production media ISP2 with the following composition: Artificial seawater (ASW) with a sea salt concentration of 30 g/L, glucose concentration of 4 g/L, yeast extract concentration of 4 g/L, and malt extract concentration of 10 g/L [14]. The flasks were then incubated for 48 h to 72 h at 25 ºC and 120 rpm (see supplementary material for detailed flow diagram of the polymer extraction process). The biomass was lyophilised and the PHA was extracted using dispersion method [14]. The biomass and polymer yield were conducted in triplicate, and the results were reported as the mean values accompanied by the standard deviation. The bar graph was constructed using GraphPad Prism 8, developed by GraphPad Software Inc. in the USA. Polymer yield was calculated using the below equation [1].

$$Polymer\,Yield\,\left( \% \right)\, = \,{{Mass\,of\,polymer\,\left( g \right)} \mathord{\left/ {\vphantom {{Mass\,of\,polymer\,\left( g \right)} {(Cell dry weight \left( g \right)}}} \right. \kern-0pt} {(Cell dry weight \left( g \right)}}\, \times \,100$$

**Table 4:** Screening of *phaC* genes and characterisation

Strain	Taxonomic assignment based on 16SRNA	Isolated from	*phaC* class I	*phaC* class II	*phaC* class III	*phaC* class V	Gram (N/P)
A9	*Bacillus hwajinpoensis*	WM	−	−	−	−	Variable
F2	*Bacillus stercoris*	PW	−	−	−	−	P
MC2	*Halomonas titanicae*	MT	+	−	−	−	N
LY	*Pseudalkalibacillus hwajinpoensis*	HI	−	−	−	−	Variable
C4	*Bacillus pacificus*	WM	−	−	+	−	P
B4	*Bacillus pacificus*	WM	−	−	+	−	P
B12	*Bacillus mycoides*	WM	−	−	+	−	P
PW3	*Bacillus pumilus*	PW	−	−	−	−	P
B9	*Pseudalkalibacillus hwajinpoensis/ Bacillus baekryungensis*	WM	−	−	−	−	Variable
NQ7	*Halomonas profundus/titanicae*	MT	+	−	−	−	N
MB2	*Marinobacter sediminum* *Marinobacter similis*	MT	+	−	−	−	N
B80	*Pseudalkalibacillus hwajinpoensis*	BR	−	−	−	−	Variable
BS1	*Pseudalkalibacillus hwajinpoensis*	MT	−	−	−	−	Variable
PW2	*Cytobacillus firmus*	PW	−	−	−	−	P or variable
DINO	*Halomonas alkaliphila*	MT	+	−	−	−	N

*WM* Weymouth, *PW* Port William, *MT* Montrose, *HI* Holy Island, *BR* Bristol, *N* Negative, *P* Positive, + PHA accumulating bacteria, − bacteria not accumulating PHA

### Nuclear magnetic resonance (NMR)

The structure of the PHA produced was determined using ^13^C and ^1^H-NMR spectroscopy on a Bruker AVNEO700 instrument, equipped with the Waltz65 decoupling program, the ZGPG30 pulse program, and the Z154039_0001 probe head. 20 mg of the purified polymer was dissolved in 1 mL of deuterated chloroform (CDCl3) before the analysis (final concentration 20 mg/mL) [55]. The ^13^C experimental parameters were set as follows: spectrometer frequency (SF01) at 176.124 MHz, temperature (TE) at 25 °C, number of scans (NS) at 1024, and pulse delay (D1) of 2 s. For the ^1^H-NMR, the SF01 was 700.35 MHz, with a TE of 25 °C, NS at 8, and a D1 of 1 s. Detailed acquisition parameters, power levels, and processing settings are provided in the supplementary material. The observed chemical shifts in parts per million (ppm) indicating the environment of ^13^C and ^1^H-atoms were used to deduce the functional groups, substructures and the full molecular structure and confirmed based on literature [41].

## Results and discussion

### Development of degenerate primers using HYDEN tool for the detection of phaC genes

In this study, the Highly Degenerate (HYDEN) tool developed by Linhart and Shamir, [27] was used to design nine degenerate primer pairs for the detection of *phaC* genes in bacteria (Table 3). A total of 65 *phaC* gene sequences from class I, 10 from class II, 19 from class III, 30 from class III/IV, and 6 from class IV were used in the design which can be found in the supplementary material. The NCBI database contains more *phaC* synthase sequences than those utilised in this study for primer design. However, we carefully selected a representative sample size based on variations in bacterial strains and *phaC* sequences. Degenerate primers are useful when studying a gene family that is only partially understood or known in a related species and have been increasingly utilised in various molecular research to discover and evaluate genes within microbial populations [8, 32, 36]. While several species may possess conserved amino acids in their proteins, differences in *phaC* sequences were observed during sequence alignments using MUSCLE in MEGA 11 due to the degeneracy of the genetic code. To account for these variations, the HYDEN tool allowed the design of primers with specified length and degeneracy, for coverage of many DNA sequences in the given set. The coverage results generated by HYDEN, shown in Table 3, indicate the number of *phaC* input sequences that align with each primer pair, allowing for up to two mismatches at both ends when combined. For example, primer phaCF1 binds to 52 out of 65, and primer phaCR1 binds to 48 out of 65 *phaC* input sequences, for a total accumulated percentage of 77% of the *phaC* gene. The degeneracy coverage column displays the number of *phaC* sequence variants that the primer can amplify.

### In silico validation of degenerate primers using De-MetaST-BLAST tool

The specificity and coverage of the nine highly degenerate primer pairs developed were validated using De-MetaST-BLAST, which allows for BLAST analysis of degenerate primers against the NCBI database, as well as the Eurofins Genomics Oligo Analysis Tool. An example of De-MetaST-BLAST in silico PCR output for phaCF3 and phaCR3 primer pair can be found in the supplementary material. Five primer combinations, phaCF1/phaCR1, phaCF3/phaCR3, phaCF5/phaCR5, phaCF8/phaCR6 and phaCF8/phaCR8 were identified as potentially suitable for amplifying *phaC* gene. Based on the top 10 BLASTx hits, these primers returned the specific *phaC* sequence. However, the primer pair phaCF2/phaCR2 and phaCF4/phaCR4 which had the highest degeneracy values, 2048/4608 and 1536/1728 respectively, returned non-specific sequences. It was noted that degeneracy plays a crucial role in determining the specificity of in silico PCR. A primer with a high degree of degeneracy may have fewer exact matches to the template, leading to non-specific hits. In contrast, primers with fewer degenerate positions, such as phaCF3/phaCR3, consistently yielded a higher success rate. This is because primers with fewer degenerate positions are more specific to the target sequence, reducing the chances of non-specific binding and resulting in more reliable amplification [11, 16, 32]. Therefore, in order to create primers that accurately target and amplify a wide range of genes, it is necessary to utilise primers with a high degree of degeneracy. Simultaneously, in order to reduce the likelihood of increasing unrelated sequences, it is necessary to clearly define and restrict degeneracy and optimise other relevant primer parameters for successful PCR [16]. The HYDEN command-line parameters used in this study can be found in the supplementary material.

### In vitro validation of degenerate primers using prototype strains

**Fig. 1:**
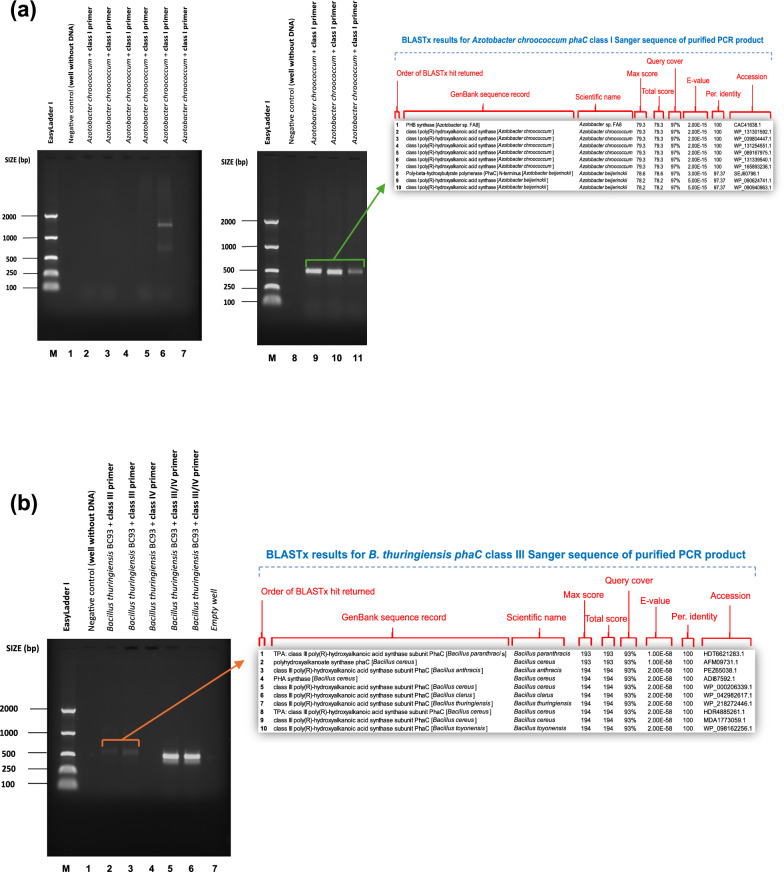

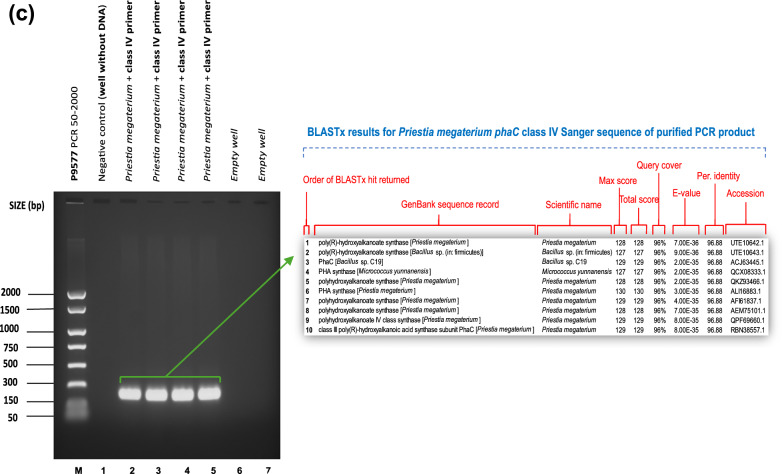
*phaC* gene amplification. Lane M: EasyLadder 1/P9577 PCR 50–2000 ladder. BLASTx search results display the top 10 sequences that have produced significant alignments for the sequenced PCR amplicons. **a**: *phaC* class I gene amplification with phaCF1 and phaCR1 primer pair using *Azotobacter chroococcum* (NCIMB10427). Annealing temperatures: 1 = 46–50 °C, 2 = 49.2 °C, 3 = 48.5 °C, 4 = 48 °C, 5 = 47.5 °C, 5 = 46.8 °C, 7 = 46 °C, 8 = 43–50 °C, 9 = 44 °C, 10 = 44 °C, 11 = 45 °C. **b** *phaC* class III & IV gene amplification with phaCF5 and phaCR5 and phaCF8 and phaCR8 primer pairs using *Bacillus thuringiensis* BC93 (NCIMB8705). Annealing temperatures: 1 = 43–48 °C, 2 = 45 °C, 3 = 46 °C. **c** *phaC* class IV gene amplification with phaCF8 and phaCR6 primer pair using *Priesta megaterium* (NCIMB8674). Annealing temperatures: 1 = 47–51 °C, 2 = 50.7 °C, 3 = 49.3.2 °C, 4 = 48.1 °C, 5 = 47.4 °C

**Fig. 2:**
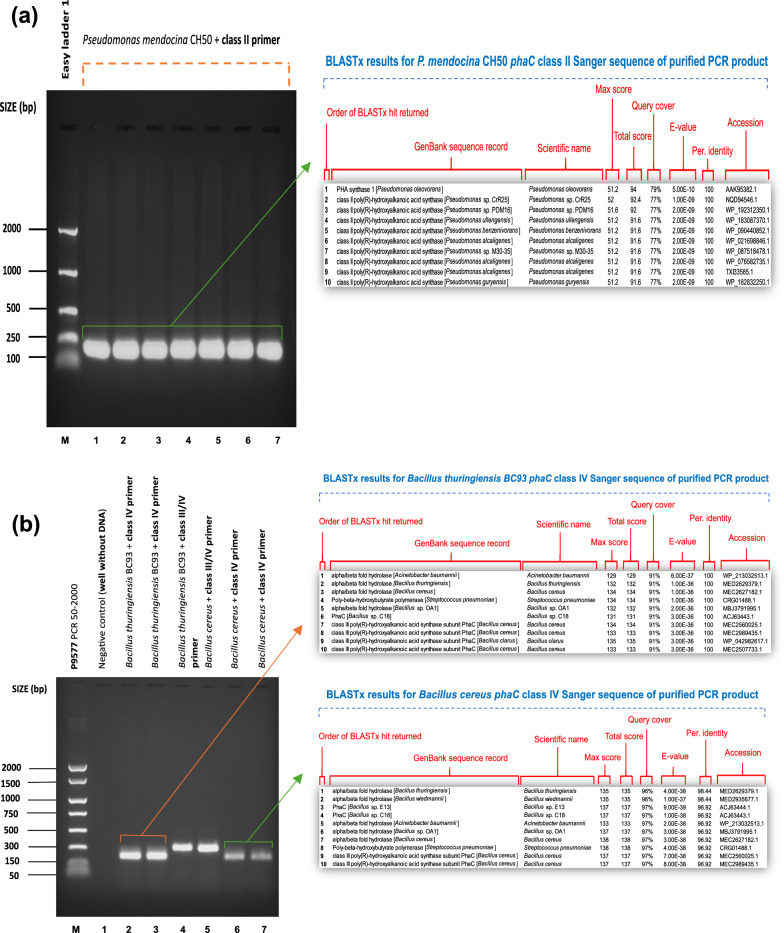

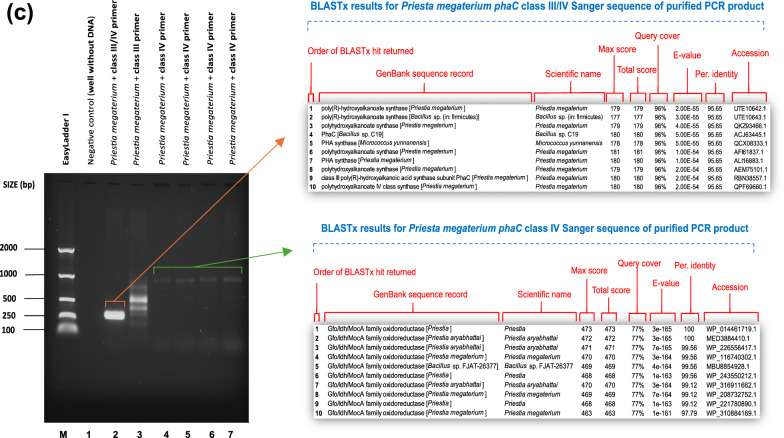
*phaC* gene amplification. Lane M: EasyLadder 1/P9577 PCR 50-2000 ladder. BLASTx search results display the top 10 sequences that have produced significant alignments for the sequenced PCR amplicons. **a** *phaC* class II gene amplification with phaCF3 and phaCR3 primer pair using *Pseudomonas mendocina* CH50 (NCIMB10541)*.* Annealing temperatures: 1 = 58.0 °C, 2 = 57.7 °C, 3 = 57.2 °C, 4 = 56.5 °C, 5 = 55.6 °C, 6 = 54.8 °C, 7 = 54.3 °C. **b** *phaC* class IV gene amplification with phaCF8 and phaCR6 primer pair (wells 2, 3, 6 and 7) and *phaC* class III/IV gene amplification using phaCF8 and phaCR8 primer pair (wells 4 and 5) using *B. thuringiensis* BC93 (NCIMB8705) and *B. cereus* (NCIMB13123). **c** *phaC* class IV gene amplification with phaCF5/phaCR5, phaCF6/phaCR6 and phaCF8/phaCR8 primer pairs using *Priesta megaterium* (NCIMB8674). Annealing temperatures: 1 = 46–50 °C, 2 = 50.0 °C, 3 = 46.0 °C, 4 = 47.5 °C, 5 = 47.0 °C, 6 = 47.0 °C, 7 = 47.0 °C

Following in silico tests, the five shortlisted primer combinations (phaCF1/ phaCR1, phaCF3/ phaCR3, phaCF5/ phaCR5, phaCF8/ phaCR6 and phaCF8/ phaCR8) were next tested in vitro using genomic DNA from already known *phaC* gene carrier species as shown in "Bacterial Strains, Maintenance and Growth Medium" section. Amplification of the expected amplicon confirmed the presence of the *phaC* gene in the reference bacteria and showed no amplification in the negative control (Figs. 1and 2). The amplicons were sequenced, and a BLASTx search was then performed on the top 10 sequences that yielded substantial alignments. The BLAST findings demonstrate that the nucleotide sequence of the *phaC* gene was successfully amplified, showing 97–100% similarity to the class I, II, III, or IV *phaC* genes from other PHA-accumulating strains. The BLAST search also lists other strains with high identity to the amplified *phaC* classes, as expected. The primer combinations phaCF2/phaCR2 and phaCF4/phaCR4 did not produce any amplicons, which is consistent with the *in-silico* findings in "In silico validation of degenerate primers using De-MetaST-BLAST tool" section. In addition to PCR, multiplex PCR was considered. However, due to the high variation in annealing temperatures between primer sets, it was deemed inapplicable.

### Screening of a novel marine bacterial strains for *phaC* gene and PHA biosynthesis

Following the successful validation of the degenerate primers, the following final four sets of primers, phaCF1/phaCR1 for class I, phaCF3/phaCR3 for class II, phaCF5/phaCR5 for class III and phaCF8/phaCR8 for class III & IV were selected to screen 15 novel marine bacterial strains sourced from UK marine sediment (Tables 4 and 5).

**Table 5:** Final set of degenerate primers used for *phaC* amplification

Primer ID	Class	Sequence* (5’-3’)	Amplicon size (bp)	Ta^y^ [˚C]
*phaCF1* *phaCR1*	I I	ATCAAYAAGTAYTACATYCT TTCCAGWASARCAKRTC	558	44
*phaCF3* *phaCR3*	II II	CTGGATGCGCCCCAACGA TCSAYCGGCGTRCCRCACA	203	58
*phaCF5* *phaCR5*	III III	GAHCATATTTCTAGCACAGA CCCCARTCNADCABRTAC	708	46
*phaCF8* *phaCR8*	III & IV III & IV	GTGTAYNTGYTDGAYTGGGG GGRATRTTKCCVARYGTATC	352 352	50

^x^Nucleotide bases other than the standard Watson–crick bases depict the universal codes for degenerate bases or "wobbles": R = A/G, Y = C/T, M = A/C, K = G/T, S = C/G, W = A/T, B = C/G/T, D = A/G/T, H = A/C/T, V = A/C/G, and N = A/C/G/T

^y^Ta = annealing temperature in °C

**Fig. 3:**
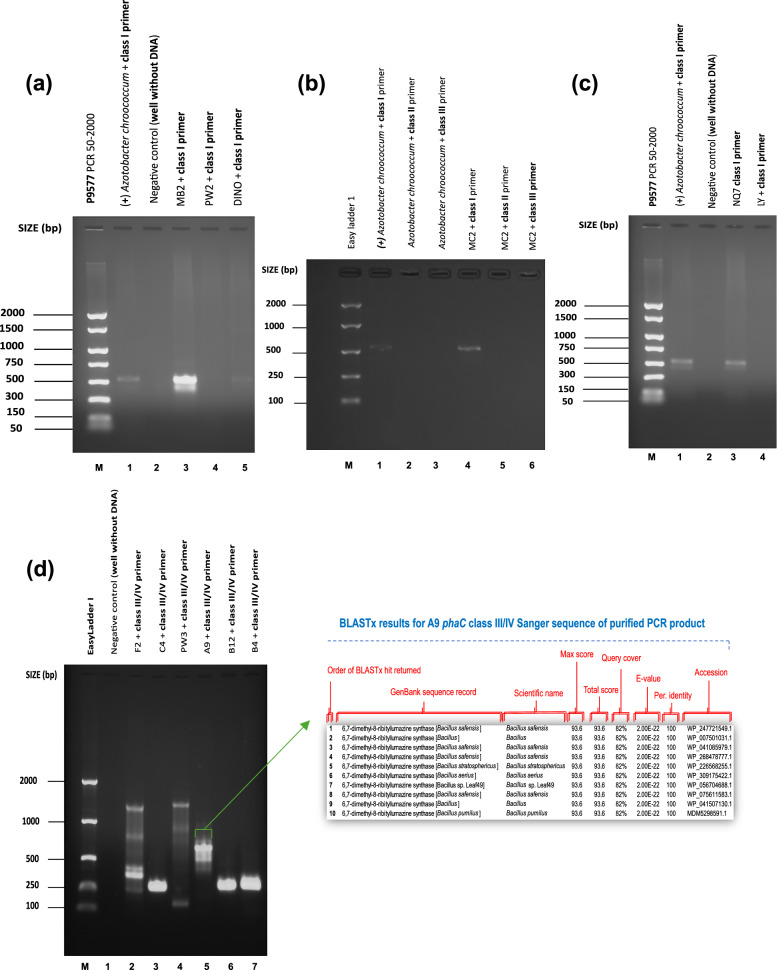
*phaC* gene amplification. Lane M: EasyLadder 1/P9577 PCR 50-2000 ladder. BLASTx search results display the top 10 sequences that have produced significant alignments for the sequenced PCR amplicons. **a** Screening *of* marine bacterial strains (*Marinobacter* sp. MB2, *Cytobacillus firmus* PW2 and *Halomonas alkaliphila* DINO) using class I primer sets (phaCF1 and phaCR1). **b** Screening of marine bacterial strain *Halomonas titanicae* MC2 for class I, II and III *phaC* gene. **c** Screening of marine bacterial strains *Halomonas profundus* NQ7 and LY for class I *phaC* gene. **d** Screening of marine bacterial strains (*Bacillus stercoris* F2, *Bacillus pacificus* C4, *Bacillus pumilus* PW3, *Bacillus hwajinpoensis* A9, *Bacillus mycoides* B12 and *Bacillus pacificus* B4) with class III/IV primer pair phaCF8 and phaCR8

Seven strains (*Halomonas alkaliphila* DINO, *Marinobacter* sp. MB2, *Halomonas profundus* NQ7, *Halomonas titanicae* MC2, *Bacillus pacificus* C4, *Bacillus pacificus* B4, and *Bacillus mycoides* B12) screened positive for the *phaC* gene. Consistent with previous studies for marine environments, the sequences found in this investigation matched PHA synthase class I and III [29, 31]. The bacterial strains used for initial validation of the PCR assay with confirmed PHA production from prior studies were used as positive control [6, 30, 38, 44, 54]. The amplicons obtained as shown in Fig. 3 were subjected to Sanger sequencing and subsequently compared to the NCBI database. The sequence identities were consistent with *phaC* genes, showing 100% identity with *phaC* genes and proteins previously deposited in the NCBI database (data not shown). However, during the screening of marine bacteria, some strains produced additional bands on the gel that were not of the expected amplicon size.

Figure 3d shows an example of non-specific amplification when *Bacillus hwajinpoensis* A9 strain was screened using class III/IV primer sets phaCF8 and phaCR8. The non-specific PCR amplicon was sequenced and identified as a gene that encodes for 6,7-dimethyl-8-ribityllumazine synthase. Thus, to use phaCF8 and phaCR8 primer set as a screening tool, we recommend matching PCR bands to the expected amplicon size, as this appears to be characteristic when using degenerate primers. A literature search indicates that few studies address degenerate primer design (DPD) problem due to the challenges of optimising PCR assays with degenerate primers compared to non-degenerate ones [11, 16].

**Fig. 4:**
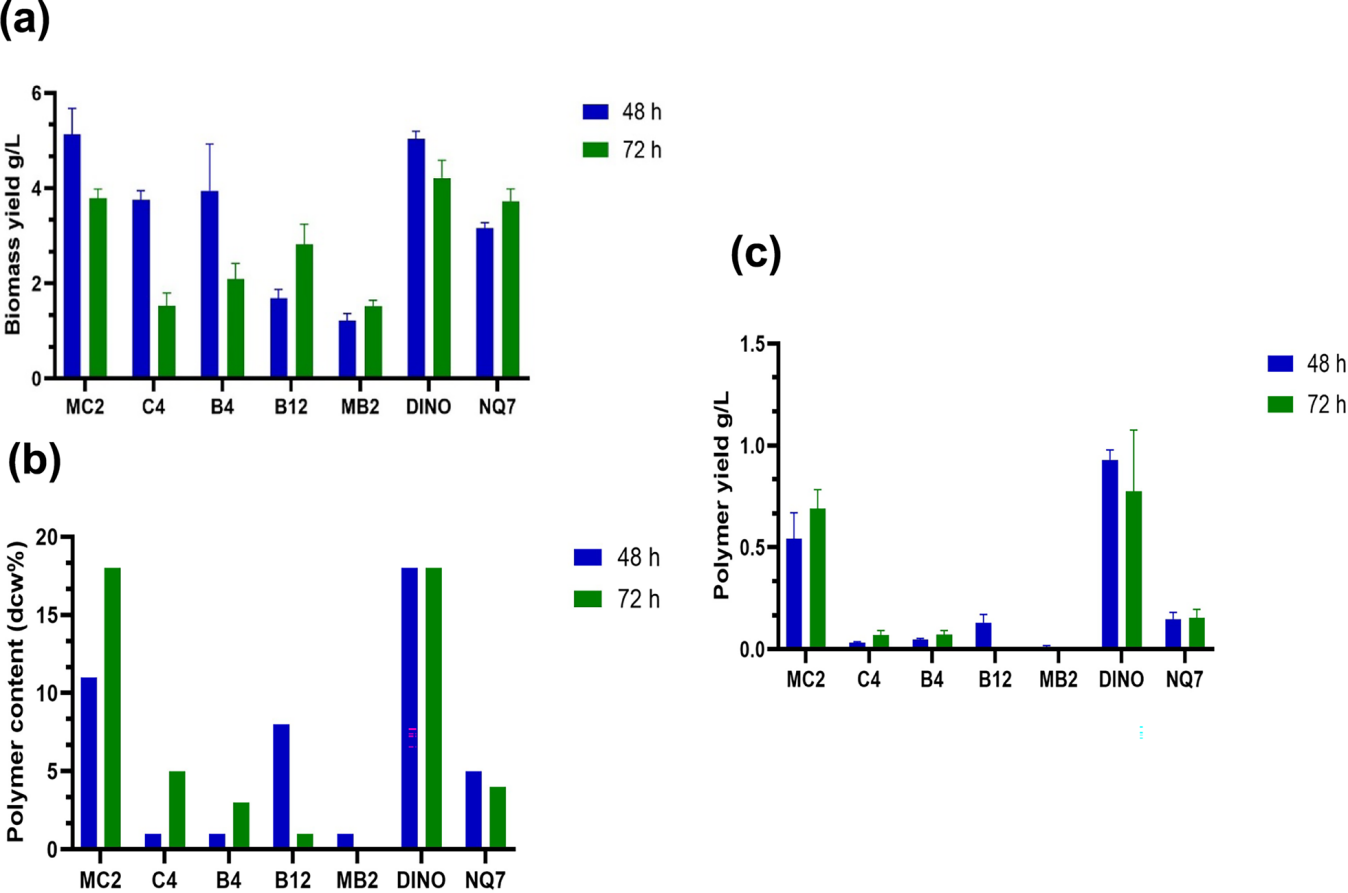
**a** Biomass (g/L), **b** polymer yield (g/L) and **c** polymer content (dry cell weight dcw %) in 48 h and 72 h of cultivations with ISP2 medium. The marine bacteria strains used were *Halomonas alkaliphila* DINO, *Marinobacter* sp. MB2, *Halomonas profundus* NQ7, *Halomonas titanicae* MC2, *Bacillus pacificus* C4, *Bacillus pacificus* B4, and *Bacillus mycoides* B12. Mean values and SD are calculated from triplicate measurements

Following the screening of 15 novel marine bacterial strains and classification of the *phaC* gene, strain identification was performed through amplification of the 16S rDNA gene, which encodes the 16S ribosomal RNA (rRNA). Both 16S rDNA and 16S rRNA exhibit a high degree of evolutionary conservation and are among the most commonly used genetic markers for studying bacterial phylogeny and identifying novel strai [26]. PCR was carried out as previously described, and the resulting amplicons were purified and Sanger sequenced by Eurofins Genomics. 16S similarity values, along with corresponding E-values are presented in (Table 6). Additionally, PHA production was carried out via flask cultivation for *phaC* screening-positive (n = 7) and *phaC* screening*-*negative (n = 8) cultures, as summarised in (Table 4). Figure 4 illustrates seven bacterial cultures—*Halomonas titanicae* MC2, *Bacillus pacificus* C4, *Bacillus pacificus* B4, *Bacillus mycoides* B12, *Marinobacter* sp. MB2, *Halomonas alkaliphila* DINO and *Halomonas profundus* NQ7—from the genera *Halomonas*, *Marinobacter*, and *Bacillus*, harvested at two time points (48 h and 72 h).

**Table 6:** Novel bacterial identification using 16S rDNA gene sequence

Strain	Taxonomic assignment	BLAST 16S rRNA similarity (%)	E-value
A9	*Bacillus hwajinpoensis*	99.67	0.0
F2	*Bacillus stercoris*	99.33	0.0
MC2	*Halomonas titanicae*	99.74	0.0
LY	*Pseudalkalibacillus hwajinpoensis*	99.32	0.0
C4	*Bacillus pacificus*	99.74	0.0
B4	*Bacillus pacificus*	99.82	0.0
B12	*Bacillus mycoides*	99.82	0.0
PW3	*Bacillus pumilus*	99.84	0.0
B9	*Pseudalkalibacillus hwajinpoensis/ Bacillus baekryungensis*	99.48	0.0
NQ7	*Halomonas profundus/titanicae*	100	0.0
MB2	*Marinobacter sediminum* *Marinobacter similis*	99.67	0.0
B80	*Pseudalkalibacillus hwajinpoensis*	99.39	0.0
BS1	*Pseudalkalibacillus hwajinpoensis*	99.49	0.0
PW2	*Cytobacillus firmus*	99.41	0.0
DINO	*Halomonas alkaliphila*	99.91	0.0

The highest yield of 0.93 g/L of PHA was achieved at 48 h by *Halomonas alkaliphila* DINO, with an intracellular content of 18% dry cell weight (dcw). These findings are consistent with previous research, which has shown that members of the genus *Halomonas* are effective PHA producers, especially under saline conditions that favour halophilic organisms [34, 42]. Koller et al*.,* (2007) found that *Halomonas boliviensis* could accumulate up to 1.0 g/L of PHA under similar conditions. However, at 72 h, the polymer yield for DINO decreased to 0.78 g/L. A possible reason could be that the bacteria were degrading the polymer, as PHA depolymerase, encoded by *phaZ* gene, is expressed simultaneously with *phaC,* resulting in the simultaneous synthesis and degradation of PHA [48, 67]. In addition, the intracellular content of 18% dcw observed in *Halomonas alkaliphila* DINO is lower than the maximum values reported for other *Halomonas* species. For instance, *Halomonas* sp. YLGW01 achieved PHA contents of up to 95% when utilising glucose or fructose as carbon sources in fed-batch fermentation [42].

The scientific literature contains plenty of papers on bacterial PHA production from various organic wastes under saline and non-saline conditions [2, 4, 5, 7, 12, 19, 61]. For example, *Bacillus* species, such as *Bacillus megaterium*, have been widely recognised for their PHA production abilities, with reports of PHA accumulation reaching up to 52% of dry cell weight under optimal conditions [39]. This suggests that the metabolic ability for PHA production is common among both halophilic and non-halophilic bacteria, highlighting their potential for industrial applications, particularly in sustainable bioplastic production. For instance, *Halomonas titanicae* MC2 exhibited a mean biomass yield of 5.135 g/L and a mean polymer yield of 0.452 g/L after 48 h (Fig. 4). After 72 h, the mean biomass yield decreased to 3.793 g/L, while the mean polymer yield increased to 0.6254 g/L. Compared to *H. alkaliphila* DINO, *H. titanicae* MC2 produced the next highest polymer yield. In contrast, *Bacillus pacificus* C4, *Bacillus pacificus* B4, and *Bacillus mycoides* B12 produced the lowest yields, with an intracellular content Ranging from 1 to 5% dcw. *Marinobacter* sp. MB2 exhibited the lowest mean polymer yield at 0.012 g/L. These strain-specific differences underscore that PHA production efficiency is highly dependent on the bacterial strain and is significantly influenced by cultivation conditions such as temperature, oxygen availability, carbon source, incubation time, and nitrogen levels, as evidenced by the variation observed across the seven strains analysed in this study. However, the PHA yields reported in this study are modest, especially considering the high amount of carbon sources fed to the cultures. It is important to note that yield optimisation was not the primary objective of this work; rather, PHA production was solely conducted to confirm the findings from the primer-based molecular screening tool and highlight any false positives. Therefore, in future studies, it is essential to develop an experimental design to enhance the production process and utilise metabolic engineering techniques to improve the strains [3, 8, 28, 45, 46, 53, 57, 61]. For example, knockout of *phaZ* and overexpressing *phaC* to enhance PHAs production [48, 67]. This will be vital in order to make PHA production more competitive.

### Characterisation of PHA produced with NMR

**Fig. 5:**
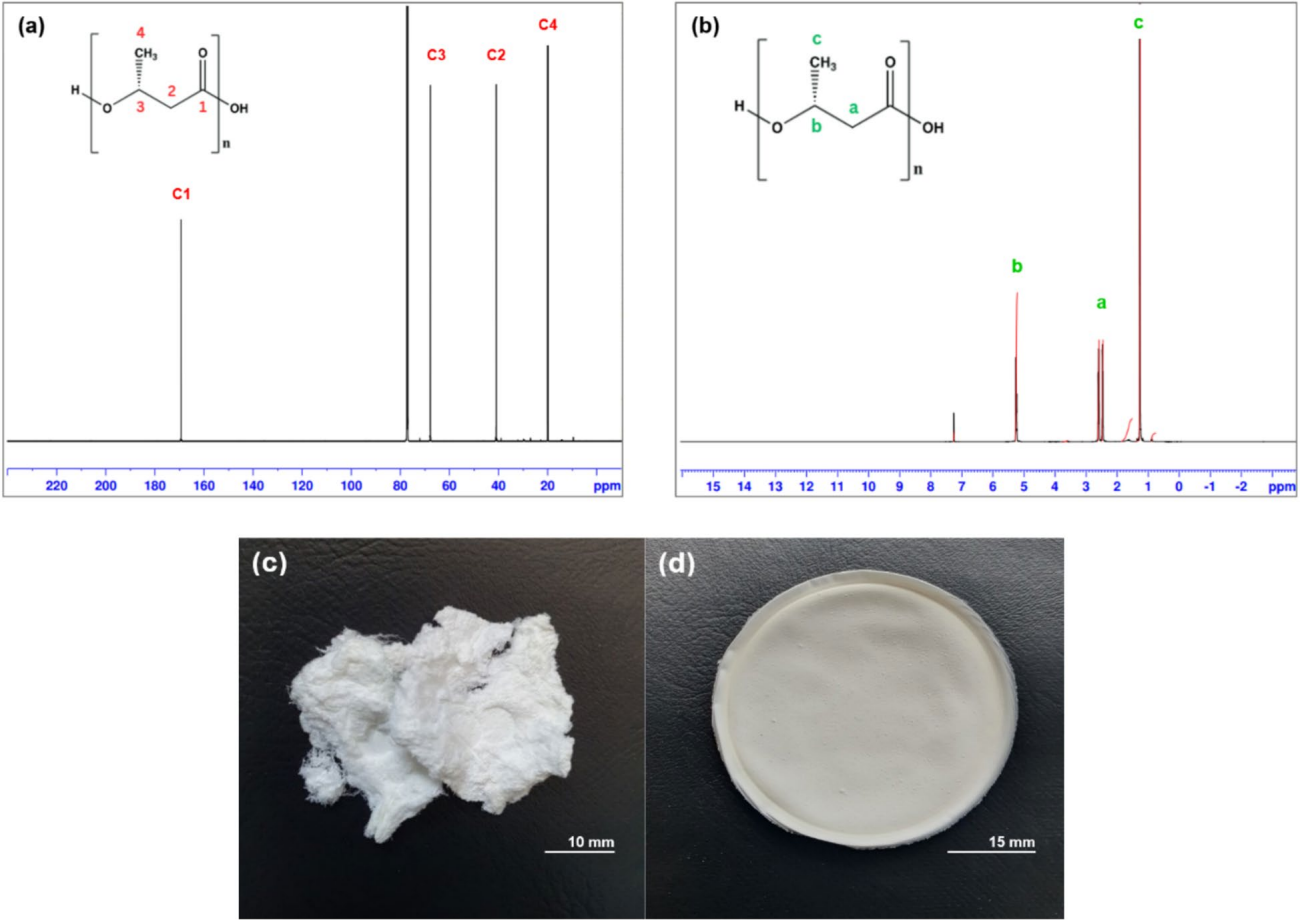
^13^C (**a**) and ^1^H-NMR (**b**) spectra of P(3HB) produced by *Halomonas alkaliphila* (DINO) using ISP2 medium. **c** P(3HB) polymer extracted from dried biomass. **d** Solvent-cast film of the P(3HB) film

^13^C and ^1^H-NMR spectroscopy were used to confirm the molecular structure of the PHA produced in ISP2 medium. All seven strains that screened positive for the presence of the *phaC* gene produced PHA, as confirmed by ^13^C and ^1^H-NMR. Figure 5 shows an example ^13^C and ^1^H-NMR spectra of P(3HB) produced by *Halomonas alkaliphila* DINO.

Three peaks were detected in ^1^H-NMR. The predominant (3xH) peak at approximately δ = 1.25 ppm corresponds to the terminal methyl (-CH3) group of the hydroxybutyrate (HB) molecule. The (2xH) peak around δ = 2.5 ppm corresponds to the methyl protons (-CH2), and the (1xH) at around 5.25 ppm corresponds to the –CH proton of the backbone of PHB. The ^13^CNMR spectrum detected the presence of 4 peaks. C1 at approximately δ = 168 suggests carbonyl groups, C2 = 41 ppm often indicates aliphatic carbons (e.g., methyl, methylene), C3 = 67 ppm suggests carbons bonded to oxygen (e.g., alcohols, ethers) and C4 = 20 ppm confirming the presence of one single monomeric unit. The monomeric composition of the polymer significantly affects its thermal and mechanical properties. Therefore, it is imperative to conduct chemical and mechanical characterisation of the produced PHA for future studies. The ^13^C and ^1^H-NMR spectra for the remaining six *phaC* screening-positive strains (*Halomonas titanicae* MC2, *Bacillus pacificus* C4, *Bacillus pacificus* B4, *Bacillus mycoides* B12, *Marinobacter* sp. MB2 and *Halomonas profundus* NQ7), confirming PHA production, are provided in the supplementary material.

## Conclusion

This study confirmed the development and validation of a highly degenerate primer-based molecular tool for identifying the main four classes of p*haC* genes in bacteria. The degenerate primers were tested both computationally against all known *phaC* sequences in the NCBI database and experimentally using known and unknown PHA-producing bacterial strains. Four primers were then shortlisted and used to screen 15 novel marine bacteria sourced from UK marine sediment and seven strains tested positive for the presence of *phaC* gene. This was further confirmed by conducting PHA production through flask cultivation for both *phaC* screening-positive (n = 7) and *phaC* screening-negative (n = 8) marine bacterial cultures, followed by characterisation using NMR. The correlation between molecular screening and PHA production outcomes reinforced the reliability of the molecular tool, confirming its potential for identifying PHA-producing bacteria in marine and terrestrial environments. E-supplementary data for this work can be found in e-version of this paper online.

## Supplementary Information

Supplementary material 1.

## Data Availability

No datasets were generated or analysed during the current study.
