# Supplementary material for: Development of a highly degenerate primer-based molecular tool for detecting and classifying the four major classes of polyhydroxyalkanoate synthase (phaC) genes in bacteria
Source: Microb Cell Fact. 2025 Sep 22;24:205. doi: 10.1186/s12934-025-02831-9 (PMC12455824; doi:10.1186/s12934-025-02831-9)
Supplement: Supplementary file 1 — Supplementary material 1. [file 12934_2025_2831_MOESM1_ESM.docx]

Figure 1. Example of De-MetaST-BLAST output for phaCF3 and phaCR3 primer set. ^x^ The search was conducted against 10 *phaC* class II gene data downloaded in FASTA format and two *in silico* amplicons recovered are shown. For hit and primer info column, the first alphanumeric character reports the hit number within a read (i.e., “1” indicates it’s the first *in silico* amplicon found within a single read). The subsequent alphanumeric characters denote the primer orientation yielding the amplicon (F = forward, R = reverse).

Table 1. showing the command-line parameters used to design the degenerate primer pair

| **Primer** | **mprimers** | **len5** | **len3** | **deg5** | **deg3** | **from5** | **to5** | **from3** | **to3** | **mis5** | **mis3** | **nentropy** | **nalgs** | **nimprove** |  |
| --- | --- | --- | --- | --- | --- | --- | --- | --- | --- | --- | --- | --- | --- | --- | --- |
|  |  |  |  |  |  |  |  |  |  |  |  |  |  |  |  |
| *phaCF1* | 1 | 20 | 20 | 30 | 30 | 0 | 1700 | -1 | -1700 | 1 | 1 | 30 | 500 | 100 |  |
| *phaCR1* | 1 | 17 | 17 | 2000 | 5000 | 0 | 1500 | -1 | -1900 | 1 | 1 | 30 | 500 | 100 |  |
| *phaCF2* | 1 | 18 | 18 | 5000 | 10000 | 1287 | 1343 | -1287 | -1343 | 1 | 1 | 30 | 500 | 100 |  |
| *phaCR2* | 1 | 20 | 20 | 5000 | 10000 | 1287 | 1343 | -1287 | -1343 | 1 | 1 | 30 | 500 | 100 |  |
| *phaCF3* | 1 | 18 | 18 | 2000 | 5000 | 300 | 1900 | -300 | -1900 | 1 | 1 | 30 | 500 | 100 |  |
| *phaCR3* | 1 | 19 | 19 | 2000 | 5000 | 1196 | 1305 | -1196 | -1305 | 1 | 1 | 30 | 500 | 100 |  |
| *phaCF4* | 1 | 20 | 20 | 2000 | 5000 | 223 | 301 | -223 | -301 | 1 | 1 | 30 | 500 | 100 |  |
| *phaCR4* | 1 | 20 | 20 | 2000 | 5000 | 100 | 1700 | -100 | -1700 | 1 | 1 | 30 | 500 | 100 |  |
| *phaCF5* | 1 | 20 | 20 | 5 | 5 | 1 | 1500 | -1 | -1500 | 1 | 1 | 30 | 500 | 100 |  |
| *phaCR5* | 1 | 18 | 18 | 200 | 200 | 100 | 1700 | -100 | -1700 | 1 | 1 | 30 | 500 | 100 |  |
| *phaCF6* | 1 | 20 | 20 | 10 | 10 | 600 | 1200 | -600 | -1200 | 1 | 1 | 30 | 500 | 100 |  |
| *phaCR6* | 1 | 23 | 23 | 10 | 10 | 1 | 1200 | -1 | -1200 | 1 | 1 | 30 | 500 | 100 |  |
| *phaCF7* | 1 | 21 | 21 | 10 | 10 | 600 | 1200 | -600 | -1200 | 1 | 1 | 30 | 500 | 100 |  |
| *phaCR7* | 1 | 20 | 20 | 10 | 10 | 1 | 300 | -1 | -300 | 1 | 1 | 30 | 500 | 100 |  |
| *phaCF8* | 1 | 20 | 20 | 100 | 100 | 1 | 1200 | -1 | -1200 | 1 | 1 | 30 | 500 | 100 |  |
| *phaCR8* | 1 | 20 | 20 | 100 | 100 | 500 | 1200 | -500 | -1200 | 1 | 1 | 30 | 500 | 100 |  |
|  |  |  |  |  |  |  |  |  |  |  |  |  |  |  |  |

Table 2. Bacterial strains used for degenerate primer design

| **Bacterial strains Accession numbers Phylum Isolation Point* phaC class** | | | | |
| --- | --- | --- | --- | --- |
| *Alcaligenes faecalis* strain ZD02  *Acidovorax avenae* subsp. avenae 19860  *Acidovorax citrulli* strain M6  *Azospirillum brasilense* strain Sp 7  *Azotobacter chroococcum* B3 | NZ_CP013119.1  NC_015138.1  NZ_CP029373.1  NZ_VISK01000003.1  NZ_CP011835.1 | Proteobacteria  Proteobacteria  Proteobacteria  Proteobacteria  Proteobacteria | Estuary sediment  Panicum miliaceum (leaf)  Symptomatic watermelon  Plant root surfaces  Soil | I  I  I  I  I |
| *Bacillus megaterium strain MS5*  *Bacillus pacificus* strain anQ-h4  *Bacillus cytotoxicus* CH_13 | KT725597.1  NZ_CP086328.1  NZ_CP024109.1 | Firmicutes  Firmicutes  Firmicutes | Soil  Ocean sediment  Vegetable puree | IV  III  III |
| *Bacillus cereus* strain FORC_047  *Bacillus tropicus* strain AOA-CPS1  *Bacillus thuringiensis* serovar berliner 10792  *Bacillus paranthracis* strain Bt C4  *Bacillus anthracis* str. 'Ames Ancestor'  *Bacillus wiedmannii* strain SR52 | NZ_CP017060.1  NZ_CP049019.1  NZ_CM000753.1  NZ_CP101135.1  NC_007530.2  NZ_CP032365.1 | Firmicutes  Firmicutes  Firmicutes  Firmicutes  Firmicutes  Firmicutes | Human blood/wound  Sea sediment  Soil  Ocean sediment  Ocean sediment  Dairy foods/environment | III  III  III  III  III  III |

Table 2. Continued.

| **Bacterial strains Accession numbers Phylum Isolation Point* phaC class** | | | | |
| --- | --- | --- | --- | --- |
| *Burkholderia oklahomensis* C6786  *Burkholderia ambifaria* strain FDAARGOS1027  *Burkholderia thailandensis* strain 2002721643  *Bacillus mobilis* 16-00177 | NZ_CP009555.1  NZ_CP066037.1  NZ_CP013411.1  NZ_FWZG01000028.1 | Proteobacteria  Proteobacteria  Proteobacteria  Firmicutes | Human  Human  Soil, rice field  Soil sample | I  I  I  III |
| *Bacillus pseudomycoides* DSM12442 | NZ_CM000745.1 | Firmicutes | Soil, Ghana | III |
| *Bacillus thuringiensis* ATCC 10792 | NZ_CM000753.1 | Firmicutes | Seashore | III |
| *Bradyrhizobium yuanmingense* strain P10 130  *Bradyrhizobium canariense* strain BTA-1  *Burkholderia stabilis* isolate E  *Burkholderia vietnamiensis* strain FL-2-3-10  *Candidatus Enterovibrio escacola* strain MJ02  *Comamonas aquatica* strain NEB418  *Cupriavidus gilardii* FDAARGOS_639 | NZ_SATS03000002.1  NZ_VSST01000013.1  NZ_LR025742.1  NZ_LOWL01000054.1  NZ_NBYY01000034.1  NZ_CP072916.1  NZ_CP054626.1 | Proteobacteria  Proteobacteria  Proteobacteria  Proteobacteria  Proteobacteria  Proteobacteria  Proteobacteria | Root nodule  Root nodule  Human, environment  Human, sputum  Lake sediment  Human  Whirlpool (USA) | I  III  I  I  I  I  I |
| *Cupriavidus metallidurans* NDB4MOL1 | NZ_FYAX01000018.1 | Proteobacteria | Wastewater plant | I |

Table 2. Continued.

| **Bacterial strains Accession numbers Phylum Isolation Point* phaC class** | | | | |
| --- | --- | --- | --- | --- |
| *Cupriavidus pauculus* BHJ32i | NZ_CP084279.1 | Proteobacteria | Human | I |
| *Cupriavidus taiwanensis* LMG 19424  *Duganella zoogloeoides* ATCC 25935  *Enterovibrio norvegicus i*solate Alg239-V16  *Sulfitobacter mediterraneus* strain SC7-37  *Grimontia hollisae* strain FDAARGOS_111  *Granulibacter bethesdensis* strain NIH7.1  *Halomonas meridiana* ACAM 239 | NC_010528.1  NZ_KB912920.1  NZ_UNRK01000018.1  NZ_CP068998.1  NZ_CP014056.2  NZ_CP018193.1  NZ_FSQX01000001.1 | Proteobacteria  Proteobacteria  Proteobacteria  Proteobacteria  Proteobacteria  Proteobacteria  Proteobacteria | Root nodule  Zoogloeal mass on limestone  Turbot gut larvae  Seawater  Human faeces  Human, granulomatous  Lake | I  I  I  I  I  I  I |
| *Halomonas titanicae* SOB56 | NZ_CP059082.1 | Proteobacteria | Seawater (RMS Titanic) | I |
| *Legionella taurinensis* strain NCTC13314  *Marinobacter nauticus* ATCC 49840 | NZ_UGOZ01000001.1  NC_017067.1 | Proteobacteria  Proteobacteria | Water  Seawater | I  I |
| *Mameliella alba* strain JL351  *Marivita cryptomonadis* CL-SK44 | NZ_NIWA01000005.1  NZ_JFKD01000007.1 | Proteobacteria  Proteobacteria | Seawater  Phytoplankton | I  I |
| *Paraburkholderia caribensis* MBA4  *Paraburkholderia aspalathi* strain R-69781 | NZ_CP012746.1  NZ_JAAGEP010000088.1 | Proteobacteria  Proteobacteria | Vertisol soil  Root nodule | I  I |

Table 2. Continued.

| **Bacterial strains Accession numbers Phylum Isolation Point* phaC class** | | | | |
| --- | --- | --- | --- | --- |
| *Phaeobacter gallaeciensis* strain P128  *Phaeobacter inhibens* strain P54  *Piscirickettsia salmonis* strain Psal-070  *Photobacterium phosphoreum* JCM 21184 | NZ_CP021047.1  NZ_CP010650.1  NZ_CP039032.1  NZ_MSCQ01000002.1 | Proteobacteria  Proteobacteria  Proteobacteria  Proteobacteria | Fish (Etelis marshi)  Seawater  Fish  Fish | I  I  I  I |
| *Priestia megaterium* strain A-3  *Priestia megaterium* strain A-2  *Priestia endophytica* strain 3617_2C  *Priestia megaterium*  *Priestia megaterium* strain A-1  *Priestia megaterium* strain FR9b  *Priestia megaterium* strain SF4  *Priestia aryabhattai* strain B-3  *Priestia aryabhattai* strain B-1  *Priestia megaterium* strain 22-2  *Pseudoalteromonas luteoviolacea* H2 | MT052026.1  MT052025.1  NZ_LWAG01000024.1  AAD05260.1  MT052024.1  MN503477.1  KY855378.1  MT052029.1  MT052027.1  NZ_NKAQ01000002.1  NZ_VIGJ01000001.1 | Firmicutes  Firmicutes  Firmicutes  Firmicutes  Firmicutes  Firmicutes  Firmicutes  Firmicutes  Firmicutes  Firmicutes  Proteobacteria | Soil  Soil  Soil  Soil  Soil  Soil  Soil  Soil  Soil  Soil  Surface seawater | IV  IV  IV  IV  IV  IV  IV  IV  IV  IV  I |
| Table 2. Continued. | | | | |
| **Bacterial strains Accession numbers Phylum Isolation Point* phaC class** | | | | |
| *Pseudoalteromonas piscicida* WCPW15003 | NZ_CP081860.1 | Proteobacteria | Surface seawater | I |
| *Pseudomonas pseudoalcaligenes* CECT 5344  *Pseudomonas mendocina* S5.2  *Pseudomonas umsongensis* CY-1  *Pseudosulfitobacter pseudonitzschiae* H46  *Pseudomonas atacamensis* strain SM1  *Pseudomonas putida* NBRC 14164 | NZ_HG916826.1  NZ_CP013124.1  NZ_CP051487.1  NZ_CP054599.1  NZ_CP070503.1  NC_021505.1 | Proteobacteria  Proteobacteria  Proteobacteria  Proteobacteria  Proteobacteria  Proteobacteria | Swimming pool water  Water, soil, sediment  Soil  Toxic marine diatom  Soil and water  Soil and water | I  II  II  I  II  II |
| *Pseudomonas monteilii* strain B5 | NZ_CP022562.1 | Proteobacteria | Soil | II |
| *Pseudomonas lactis* strain SS101 | NZ_CM001513.1 | Proteobacteria | Bovine raw milk | II |
| *Pseudomonas mediterranea* strain DSM 16733 | NZ_LT629790.1 | Proteobacteria | Tomato plant | II |
| *Pseudomonas pseudoalcaligenes* CECT 5344 | NZ_HG916826.1 | Proteobacteria | Swimming pool water | II |
| *Pseudomonas poae* strain LMG 21465 | NZ_LT629706.1 | Proteobacteria | Phyllosphere of grasses | II |
| *Pseudomonas yamanorum* strain LBUM636 | NZ_CP012400.2 | Proteobacteria | Soil | II |
| *Pseudomonas nitroreducens* strain WS 5012 | NZ_JAAQXT010003.1 | Proteobacteria | Soil | I |
| *Pseudoalteromonas luteoviolacea* strain H2 | NZ_VIGJ01000001.1 | Proteobacteria | Surface seawater | I |
| Table 2. Continued. | | | | |
| **Bacterial strains Accession numbers Phylum Isolation Point* phaC class** | | | | |
| *Paraburkholderia fungorum* strain ATCC | NZ_CP010026.1 | Proteobacteria | Human | I |
| *Paraburkholderia tropica* strain IAC135 | NZ_CP049134.1 | Proteobacteria | Sugarcane roots | I |
| *Paraburkholderia hospita* strain DSM 17164 | NZ_CP026106.1 | Proteobacteria | Soil and water samples | I |
| *Polynucleobacter asymbioticus* QLW | NC_009379.1 | Proteobacteria | Lake Kasumigaura | I |
| *Plesiomonas shigelloides* strain 7A | NZ_CP087711.1 | Proteobacteria | Dog faeces | I |
| *Photobacterium iliopiscarium* strain NCIMB | NZ_PYLU01000005.1 | Proteobacteria | A pyloric ceca of herring | I |
| *Photobacterium kishitanii* strain ANT-2200 | NZ_LN794353.1 | Proteobacteria | Deep water fish | I |
| *Photobacterium carnosum* strain TMW2.2147 | NZ_WMDN01000009.1 | Proteobacteria | Poultry meat | I |
| *Priestia flexa* strain SSAI1 | NZ_CP060274.1 | Firmicutes | Soil | IV |
| *Qipengyuania flava* strain 21-3 | NZ_CP032228.1 | Proteobacteria | Seawater | I |
| *Ralstonia solanacearum* strain UW251 | NZ_CP088237.1 | Proteobacteria | Crop plants | I |
| *Ralstonia pickettii* isolate MGYG-HGUT-01384 | NZ_CABKQE010000003.1 | Proteobacteria | Human | I |
| *Rhizobium phaseoli* strain R650 | NZ_CP013532.1 | Proteobacteria | Garden beans | I |
| *Rhizobium lentis* strain BLR27 | NZ_CP071458.1 | Proteobacteria | Root nodules of lentil | III |
| *Rhodopseudomonas palustris* RCB100 | NZ_CP066699.1 | Proteobacteria | Small pond | I |
| Table 2. Continued. | | | | |
| **Bacterial strains Accession numbers Phylum Isolation Point* phaC class** | | | | |
| *Ruegeria atlantica* CECT4292 | NZ_CYPU01000006.1 | Proteobacteria | Marine sediments | I |
| *Salinivibrio kushneri* strain AL184 | NZ_CP040021.1 | Proteobacteria | Salt pan | I |
| *Solemya velum* gill symbiont NC-DML14 | NZ_MPRU01000016.1 | Proteobacteria | Subtidal sediments | III |
| *Stenotrophomonas maltophilia* NCTC10258 | NZ_LS483377.1 | Proteobacteria | Water (lake, river) | III |
| *Stenotrophomonas rhizophila* DSM 14405 | NZ_CP007597.1 | Proteobacteria | Root and stem | III |
| *Stenotrophomonas lactitubi* strain YR347 | NZ_FZPB01000001.1 | Proteobacteria | Root and stem | III |
| *Sphingobium yanoikuyae* strain S72 | NZ_CP023741.1 | Proteobacteria | Human clinical specimen | I |
| *Sphingomonas melonis* C3 | NZ_AQUJ01000001.1 | Proteobacteria | Plant Cucumis melo var | I |
| *Tritonibacter mobilis* F1926 | NZ_CP015231.1 | Proteobacteria | Seawater | I |
| *Vibrio owensii* XSBZ03 | NZ_CP019960.1 | Proteobacteria | Marine natural habitat | I |
| *Vibrio fluvialis* strain ATCC | NZ_CP014034.2 | Proteobacteria | Human faeces | I |
| *Vibrio lentus* strain 10N.261.52.F12 | NZ_MCXR02000002.1 | Proteobacteria | Cultivated oyster | I |
| *Vibrio navarrensis* strain 0053-83 | NZ_CP051120.1 | Proteobacteria | Seawater | I |
| *Variovorax paradoxus* NBRC 15149 | NZ_BCUT01000001.1 | Proteobacteria | Soil | I |
| *Xanthomonas vesicatoria* 35937 LMG911 | NZ_CP018725.1 | Proteobacteria | Lycopersicon esculentum | III |
| \| Table 2. Continued. \| \| --- \| \| **Bacterial strains Accession numbers Phylum Isolation Point* phaC class** \| | | | | |
| *Xanthomonas translucens* pv. undulosa XtLr8 | NZ_CP063993.1 | Proteobacteria | Hordeum vulgare | III |
| *Xanthomonas sacchari* strain CFBP4641 | NZ_MDEK01000003.1 | Proteobacteria | Diseased sugarcane | III |

* Isolation point details were downloaded from Bac Dive database accessible from (<https://bacdive.dsmz.de/isolation-sourc>


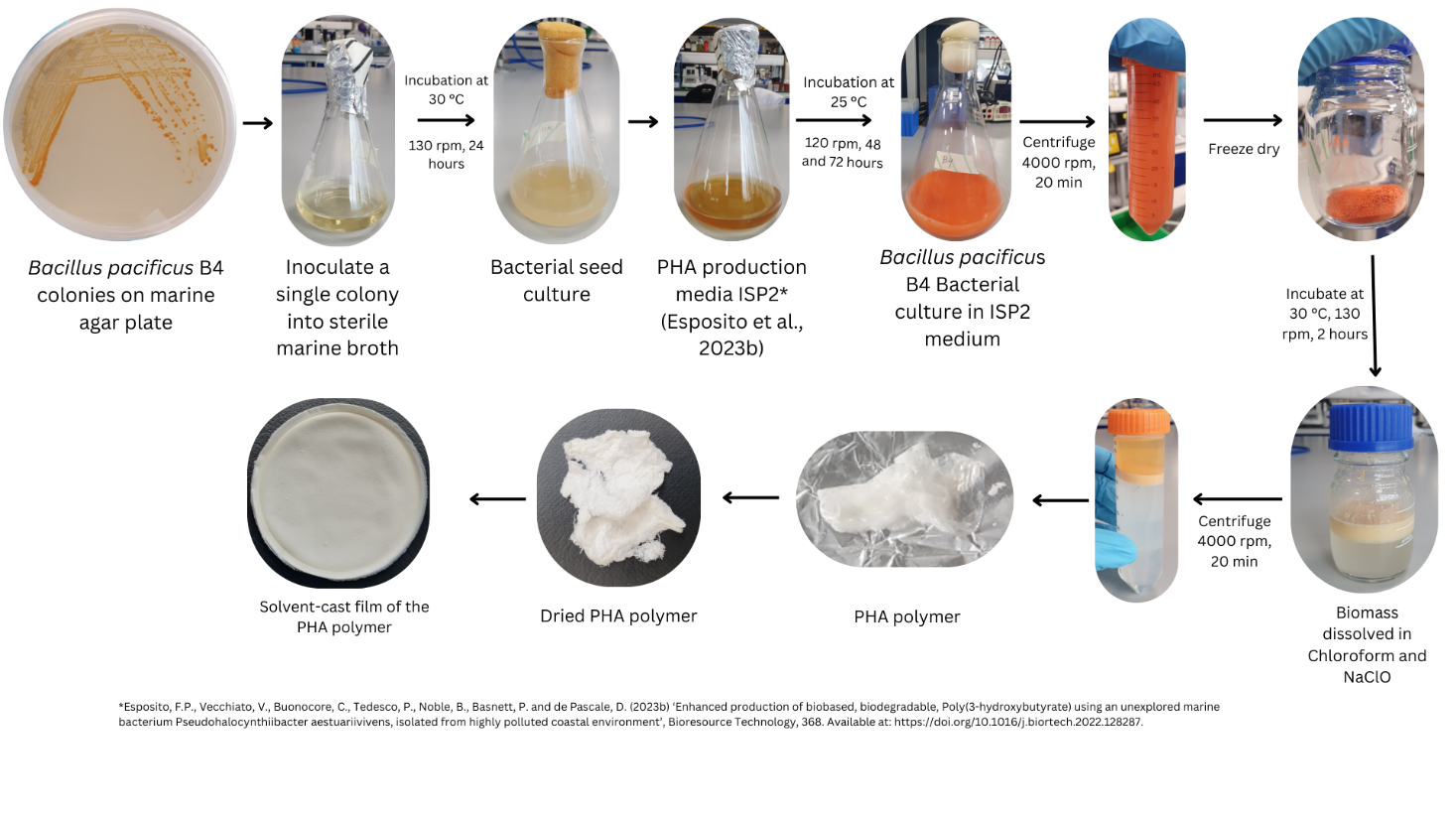


Figure 2. Showing summary of PHA extraction process


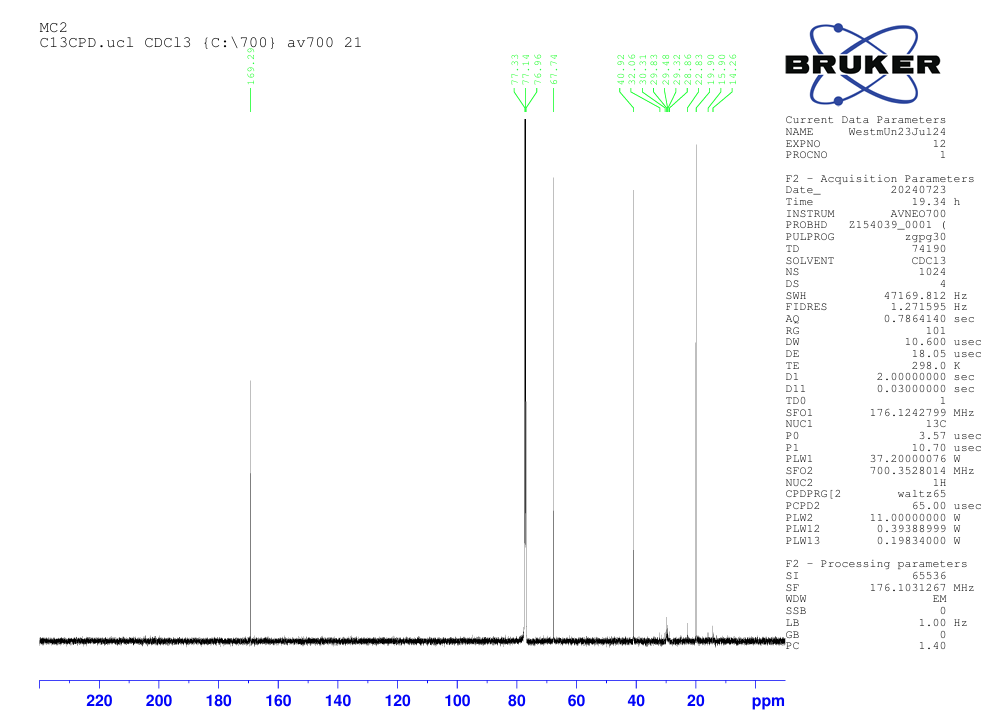

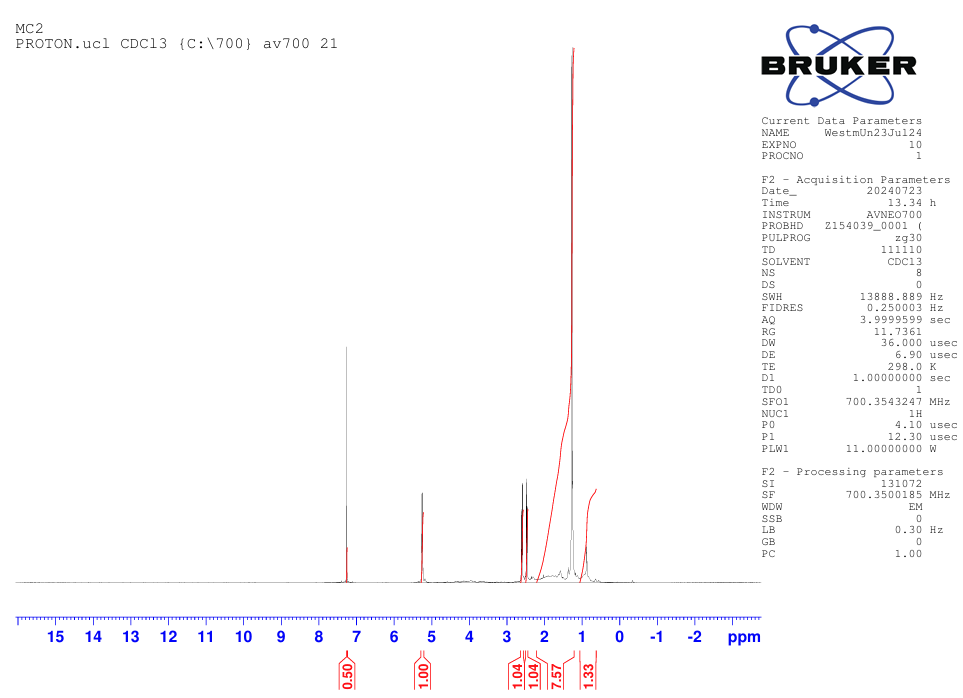

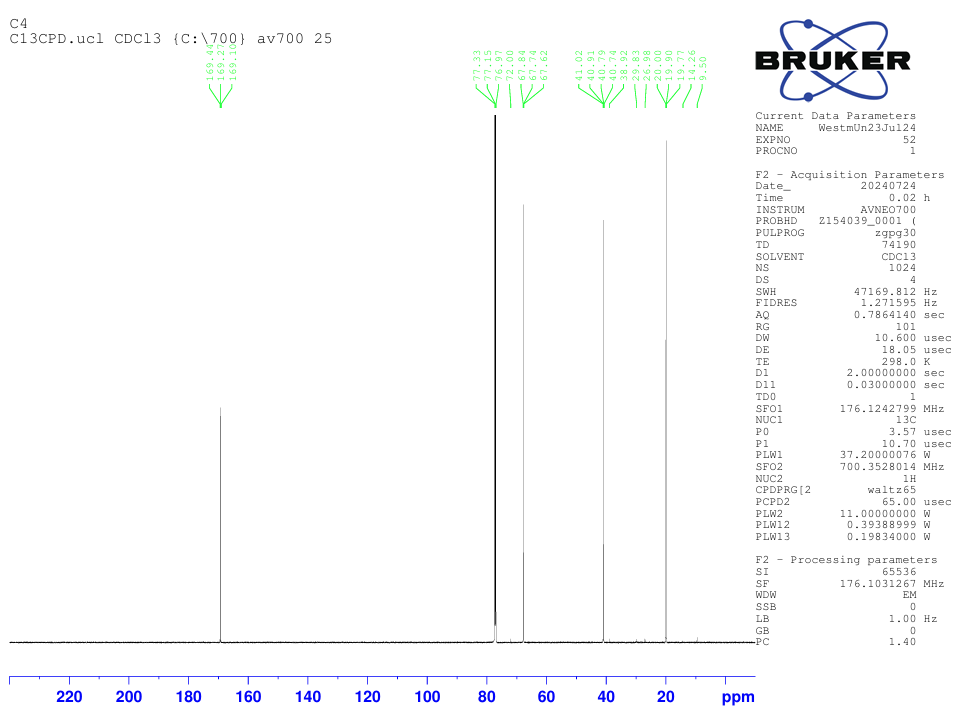

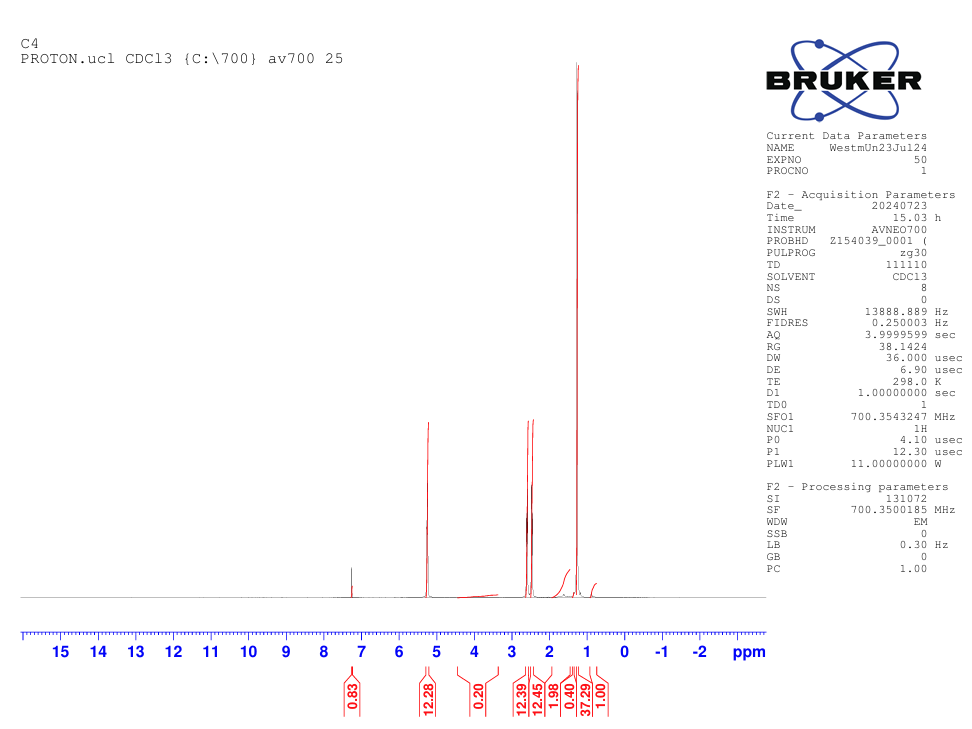

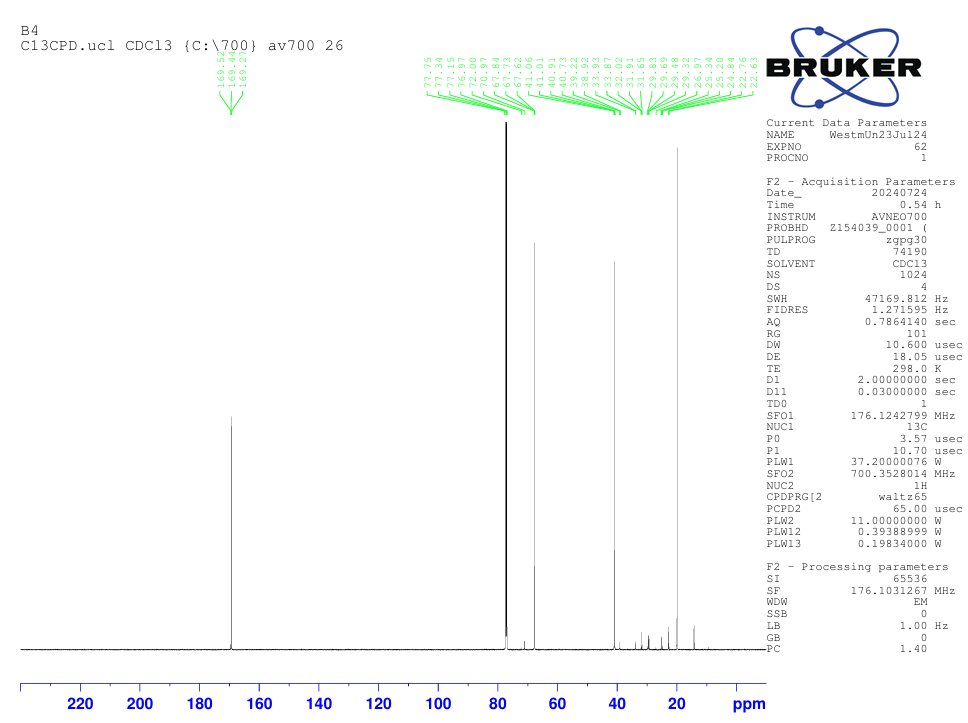

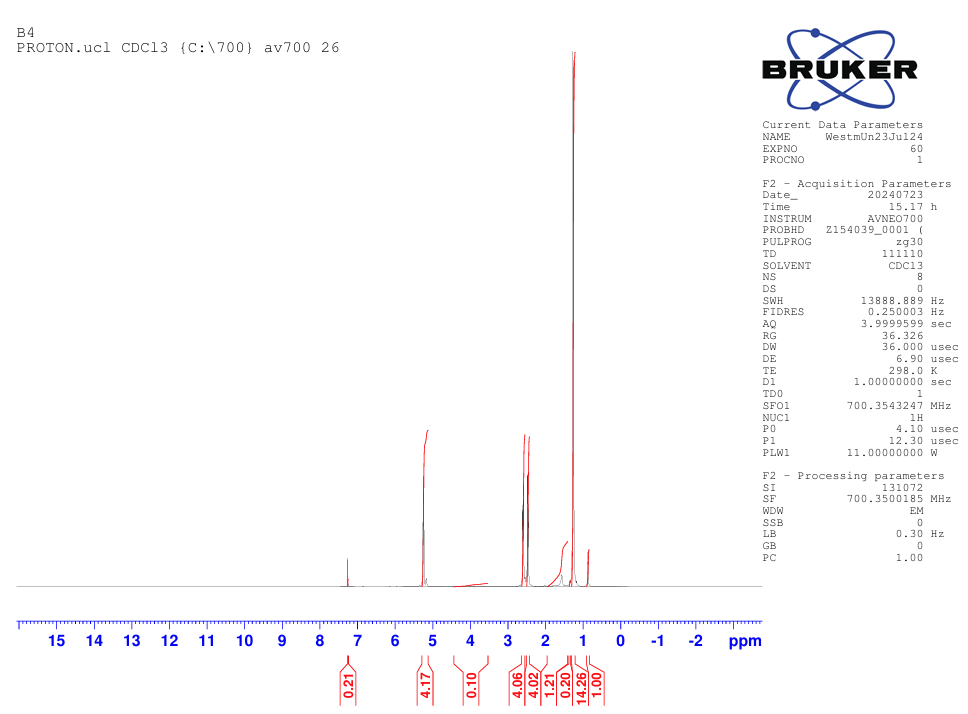

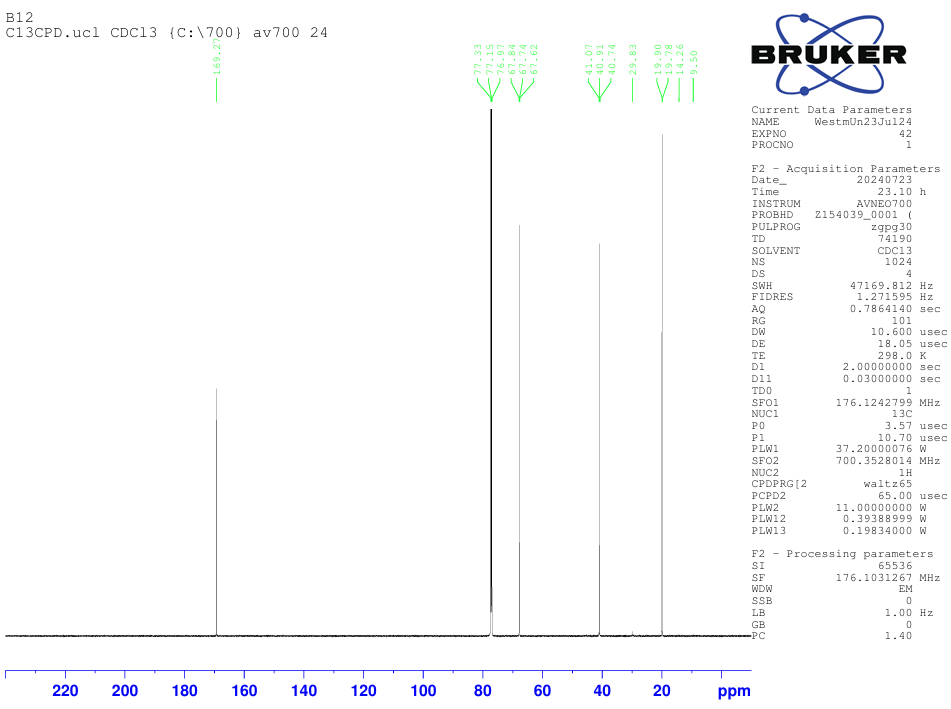

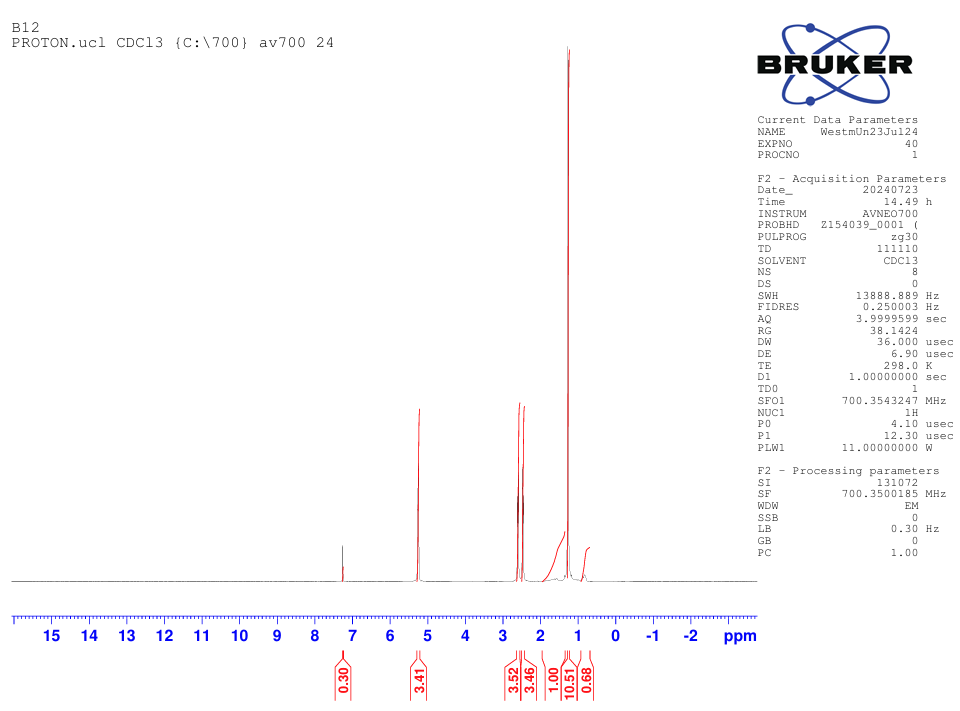

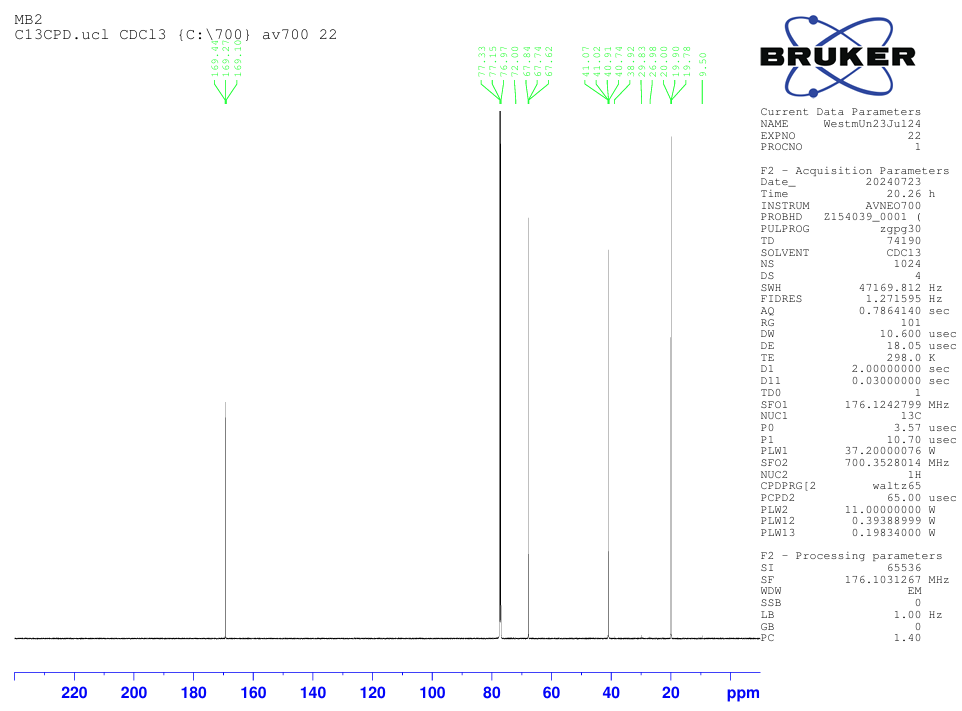

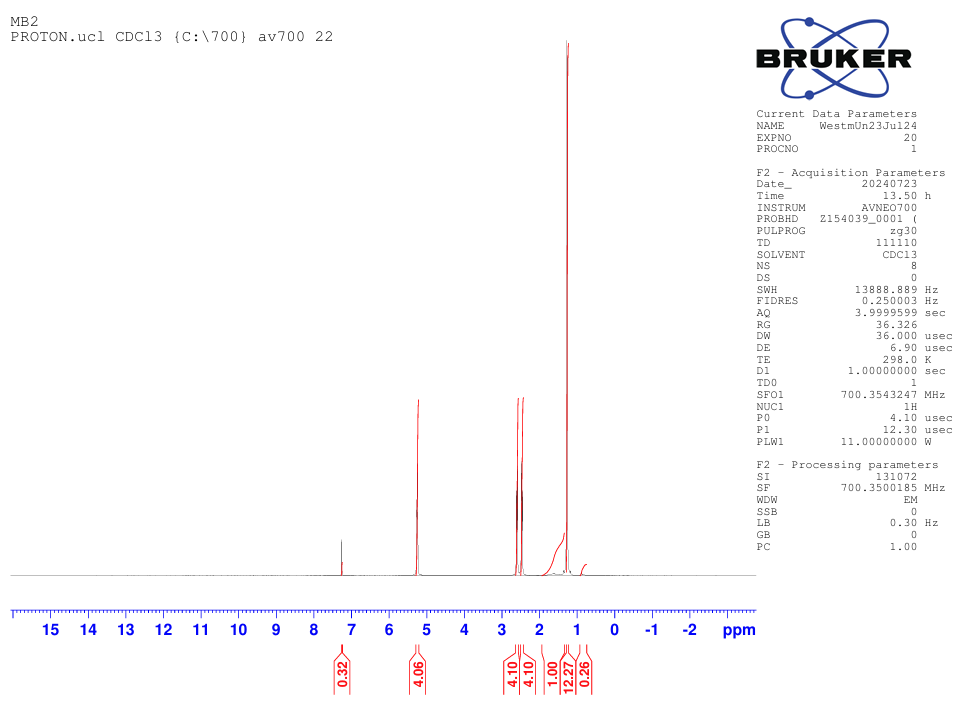

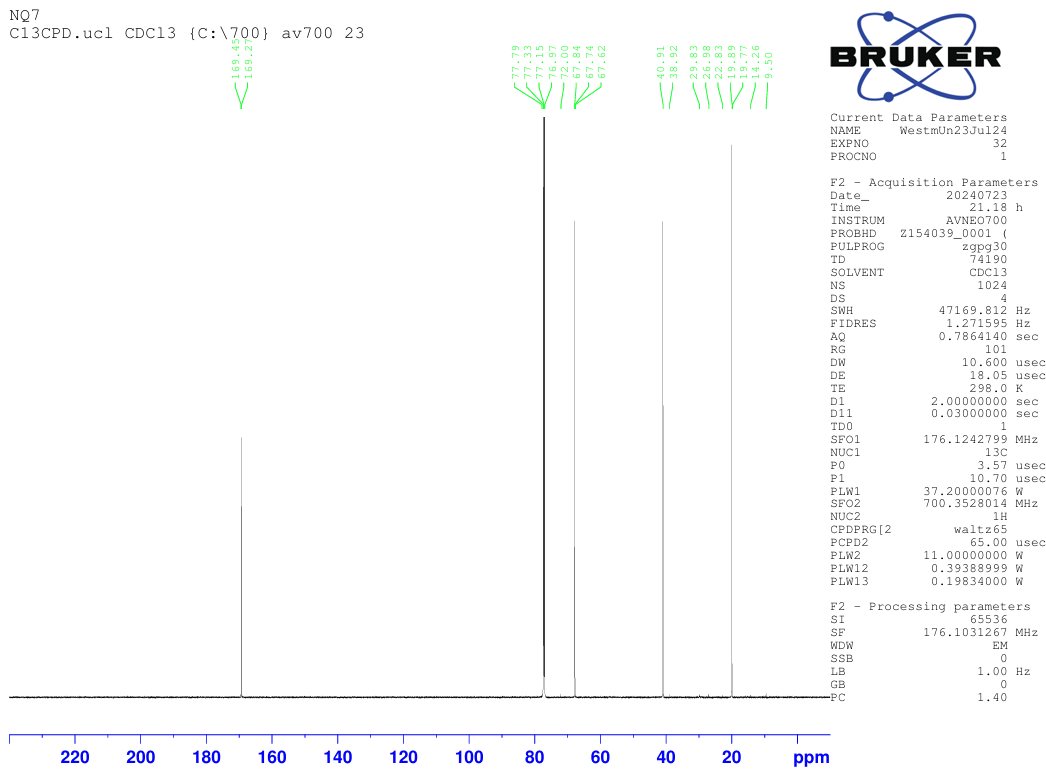

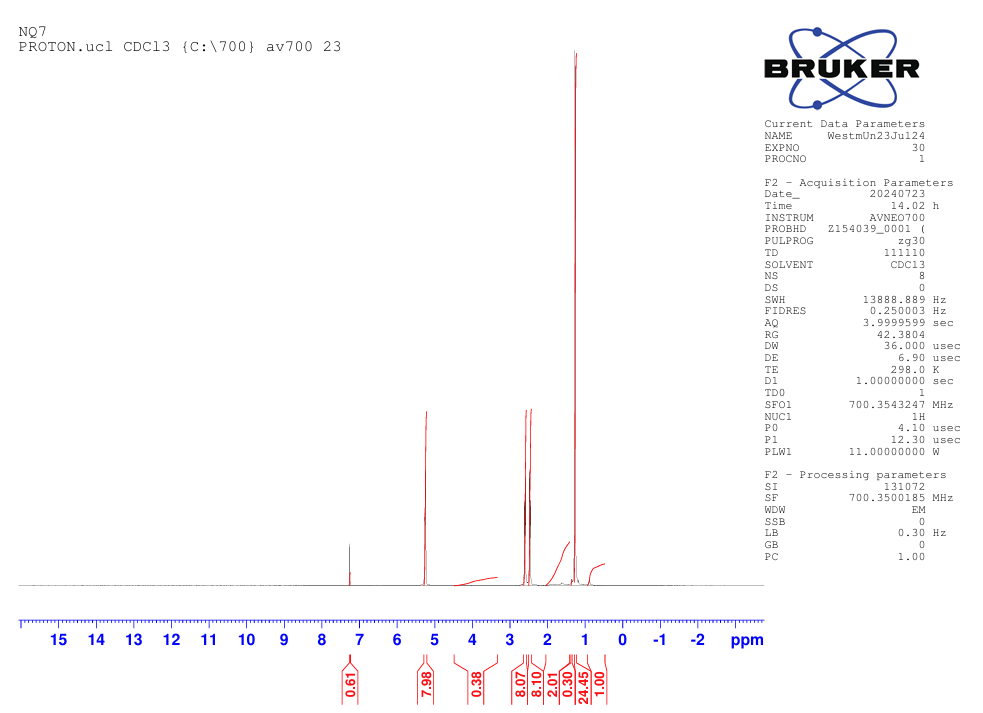


**(h)**

**(g)**

**(f)**

**(e)**

**(c)**

**(d)**

**(b)**

**(a)**

**(j)**

**(i)**

**(l)**

**(k)**

**^Notes:^** TD (Time Domain Points), SW (Spectral Width), FIDRES (Resolution), AQ (Acquisition Time), DW (Dwell Time), DE (Dead Time), RG (Receiver Gain), NS (Number of Scans), DS (Dummy Scans), D1 (Pulse Delay), TE (Temperature), PLW1 (¹³C pulse power), PLW2 (¹H decoupling), SI (Size of FT), SF (Spectrometer Freq), WDW (Window Function), SSB (Baseline Corr.), LB (Line Broadening), GB (Gaussian Factor), PC (Phase Correction)

Figure 3. Showing ^13^C and ^1^H-NMR spectra of P(3HB) produced by (**a**,**b**) *Halomonas* *titanicae* MC2, (**c**,**d**) *Bacillus pacificus* C4, (**e**,**f**) *Bacillus* *pacificus* B4, (**g**,**h**) *Bacillus mycoides* B12, (**i**,**j**) *Marinobacter* sp. MB2, and (**k**,**l**) *Halomonas* *profundus* NQ7 using ISP2 medium

Table 3. Showing selected sequences used to validate *phaC* gene PCR amplicons

| **Strain** | **Taxonomic assignment based on 16SRNA** | **Sanger DNA sequence (Forward)** | **Sanger DNA sequence (Reverse)** |
| --- | --- | --- | --- |
| CH50 | *Pseudomonas mendocina* | TCAACAACTACCTGCTCGGCAACGGAGCCAGCCGGTGTTCGACATTCTCTACTGGAACAACGACAACCACACGCCTGCCGGCTGCCCTGCACGGCGAGCTCATCGAATTGTTCCAGACCAACCCGTTGACCCGTCCCGGCGCGCTGGAAGTGTGCGGCACGCCGATCGAAAACGGCGCGCTGTGA | AATCGAAGCTCGCCATGGCAAGGCGCAGCGCGGCAGGCGTGTGGGTGTCGTTGTTCCAGTAGAGAATGTCGAACACCGGCGGCTCGTTGCCCGAGCAGGTAGTTGTTGACCCAGTAGTTCCAGATCAGGTCGTTGGGGCGCATCCAGAAGATGTC |
| *BC93* | *Bacillus thuringiensis* | AAAAGTAATTTGAAATTTGATGATTTCGTGTTTGATTATATTGCAAAAGCAGTAAAAAAATAATGCGAACTGCAAAATCGGACGAGATTTCTTTACTTGGTTATTGCATGGGTGGAACGCTAACTTCTATTTATGCAGCACTTCATCCGCACATGCCAATTCGTAATTTAATTTTCATGACAAGTCCTTTTGATTTCTCTGAAACAGGATTGTATGGTCCTTTGTTAGATGAGAAATACTTCAATTTAGATAAAGCGGTTGATACGCTCGGCA | CATGGAGTATTTCTCATCTAACAAAGGACCATACAATCCTGTTTCAGAGAAATCAAAAGGACTTGTCATGAAAATTAAATTACGAATTGGCATGTGCGGATGAAGTGCTGCATAAATAGAAGTTAGCGTTCCACCCATGCAATAACCAAGTAAAGAAATCTCGTCCGATTTTGCAGTTCGCATTACTTTTTTTACTGCTTTTGCAATATAATCAAACACGAAATCATCAAATTTCAAATGACTATCTTCTAAACCAAATGTGCCCCAATCCAGCCACATACACAAA |
| *-* | *Bacillus cereus* | TTCGTAAAAGTCTTTGAAATTTGATGATTTCGTGTTTGATTATATTGCAAAAGCAGTAAAAAAAGTAATGCGAACTGCAAAATCGGACGAGATTTCTTTACTTGGTTATTGCATGGGTGGAACGCTAACTTCTATTTATGCAGCGCTTCATCCGCACATGCCAATTCGTAATTTAATTTTCATGACAAGTCCTTTTGATTTCTCTGAAACAGGATTGTATGGTCCTTTATTAGATGAGAATACTTCAATTTAGATAAAGCGGTTGATACGCTCGGCAACATCCCAA | TTAATGGAGTTTTCTCATCTAATAAAGGACCATACAATCCTGTTTCAGAGAAATCAAAAGGACTTGTCATGAAAATTAAATTACGAATTGGCATGTGCGGATGAAGCGCTGCATAAATAGAAGTTAGCGTTCCACCCATGCAATAACCAAGTAAAGAAATCTCGTCCGATTTTGCAGTTCGCATTACTTTTTTTACTGCTTTTGCAATATAATCAAACACGAAATCATCAAATTTCAAATGACTATCTTCTAAACCAAATGTGCCCCAATCCACCACATACACAAAT |
| *-* | *Priestia megaterium* | GAAAGGCAATATGAAGCTAGATGATTATATTGTAGATTATATTCCAAAAGCGGCGAAAAAGGTGCTTCGCACTTCGAAATCTCCTGATTTGTCTGTTCTTGGTTACTGCATGGGCGGAACTATGACATCTATTTTTGCTGCATTAAATGAAGACTTGCCGATTAAAAACTTAATTTTTATGACAAGTCCATTTGATTTTTCGGATACAGGTTTATACGGAGCATTTTTAGACGATCGCTACTTTAATTTAGATAAAGCAGTAGATACGCTCGCCCAACATCCCACAC | CCGGTTAATAAAGGTAGCGAATCGTCTAAAATGCTCCGTATAAACCTGTATCCGAAAAATCAAATGGGACTTGTCATAAAAATTAAGTTTTTAATCGGCAAGTCTTCATTTAATGCAGCAAAAATAGATGTCATAGTTCCGCCCATGCAGTAACCAAGAACAGACAAATCAGGAGATTTCGAAGTGCGAAGCACCTTTTTCGCCGCTTTTGGAATATAATCTACAATATAATCATCTAGCTTCATATTGCTGTCTTCAAGCCCAGGAGTTCCCCAGTCCAGCCACGTACACT |
| MC2 | *Halomonas titanicae* | GGAATTCATGGGTGAAGTGGCTGGTGGGATCAGGGCCATACGGTGTTCCTGATCTCCTGGCGTAATCCTGGCCCGGAGCAGCGCGATATTACCTGGGCCGACTATATGCAGATGGGGCCGATCAGCGCGATAGAAGCTATCGAGCAGGCCTGCGGCGAGAAGTCGGTTAATCTCCTCAGCTACTGTGTCGGCGGCACGTTAACGGCATCTACGGTAGCGTACCTCACCAGCACCCGCCGTGGGCGCAAAGTAAAGTCGGTGACCTATATGGCCACGCTGCAGGATTTTCGTGATCCTGGCGATATCGGCGTATTTCTCAATGAGCGGGTGGTTGAAGGGATCGAACAAACGCTAGAAGCGAAGGGCTACCTTGATGGCCGCTCGATGGCTTACACTTTCAATTTGTTGCGTGAGAATGATCTGTTTTGGTCGTTCTACATTAATAACTATTTGAAGGGCGAAACGCCTGCCGCCTTTGACCTGCTCTACTGGAA | TAGGTTTAATTAATGTAGGAACGAACCAAAACAGATCATTCTCACGCAACAAATTGAAAGTGTAAGCCATCGAGCGGCCATCAAGGTAGCCCTTCGCTTCTAGCGTTTGTTCGATCCCTTCAACCACCCGCTCATTGAGAAATACGCCGATATCGCCAGGATCACGAAAATCCTGCAGCGTGGCCATATAGGTCACCGACTTTACTTTGCGCCCACGGCGGGTGCTGGTGAGGTACGCTACCGTAGATGCCGTTAACGTGCCGCCGACACAGTAGCTGAGGAGATTAACCGACTTCTCGCCGCAGGCCTGCTCGATAGCTTCTATCGCGCTGATCGGCCCCATCTGCATATAGTCGGCCCAGGTAATATCGCGCTGCTCCGGGCCAGGATTACGCCAGGAGATCAGGAACACCGTATGGCCCTGATCCACCAGCCACTTCACCATGGAATTATCTTCACGCAGATCGAGGATGTAATACTTGTTGATA |
